# The dual role of CXCL9/SPP1 polarized tumor-associated macrophages in modulating anti-tumor immunity in hepatocellular carcinoma

**DOI:** 10.3389/fimmu.2025.1528103

**Published:** 2025-03-31

**Authors:** Yu Gu, Zhihui Zhang, Hao Huang, Wenyong Zhu, Hongjia Liu, Rongxin Zhang, Nan Weng, Xiao Sun

**Affiliations:** ^1^ State Key Laboratory of Digital Medical Engineering, School of Biological Science and Medical Engineering, Southeast University, Nanjing, China; ^2^ College of Acupuncture-Moxibustion and Tuina, Nanjing University of Chinese Medicine, Nanjing, China

**Keywords:** hepatocellular carcinoma, tumor-associated macrophages, macrophage polarity, regulatory mechanisms, biomarker

## Abstract

**Introduction:**

The main challenge for cancer therapy lies in immuno-suppressive tumor micro-environment. Reprogramming tumor-associated macrophages (TAMs) into an anti-tumor phenotype is a promising strategy.

**Methods:**

A comprehensive analysis by combing multi-regional single-cell, bulk and spatial transcriptome profiling with radiomics characterization was conducted to dissect the heterogeneity of TAMs and resolve the landscape of the CXCL9:SPP1 (CS) macrophage polarity in HCC.

**Results:**

TAMs were particularly increased in HCC. SPP1^+^ TAMs and CXCL9^+^ TAMs were identified as the dominant subtypes with different evolutionary trajectories. SPP1^+^ TAMs, located in the tumor core, co-localized with cancer-associated fibroblasts to promote tumor growth and further contributed to worse prognosis. In contrast, CXCL9^+^ TAMs, located in the peritumoral region, synergized with CD8^+^ T cells to create an immunostimulatory micro-environment. For the first time, we explored the applicability of CS polarity in HCC tumors and revealed several key transcription factors involved in shaping this polarity. Moreover, CS polarity could serve as a potential indicator of prognostic and micro-environmental status for HCC patients. Based on medical imaging data, we developed a radiomics tool, RCSP (Radiogenomics-based CXCL9/SPP1 Polarity), to assist in non-invasively predicting the CS polarity in HCC patients.

**Conclusion:**

Our research sheds light on the regulatory roles of SPP1^+^ TAMs and CXCL9^+^ TAMs in the micro-environment and provides new therapeutic targets or insights for the reprogramming of targeted macrophages in HCC.

## 1 Introduction

Liver cancer ranks among the top five causes of cancer-related mortality globally (1). Hepatocellular carcinoma (HCC) is the predominant pathological subtype, which accounting for approximately 90% of liver cancer cases (2). Despite the surgery being the most effective treatment for HCC, elusive onset and swift progression often result in patients presenting too late for surgical intervention (3). Immunotherapy has transformed the landscape of cancer treatment, offering new hope for HCC patients (4). However, the effectiveness of immunotherapy is limited to a small fraction of HCC patients, highlighting the critical need to identify factors hindering immune infiltration and develop combination strategies to improve the prognosis (5).

HCC is composed of a complex tumor micro-environment (TME) consisting of cellular (tumor-infiltrating immune cells and stromal cells), chemical (cytokines and chemokines), and extracellular matrix components (6, 7). Compared to tumor cells, TME components exhibit greater genetic stability, making them more suitable targets for therapeutic intervention (8). Macrophages, acting as key components of the immune system, are extensively present in numerous tissues. Tumor-associated macrophages (TAMs) are a subset of macrophages stem from circulating monocytes and accumulate within tumors, which are closely linked to patient survival and correlate with drug resistance (9). Their roles within the TME guide the mutual evolution of cancer ecosystem during the stages of tumor growth, spread, and reaction to treatment (10). Therefore, TAMs have emerged as an attractive therapeutic target.

Numerous studies have utilized single-cell RNA sequencing (scRNA-seq) to dissect the diverse functional subsets of TAMs (11–13). However, TAMs are thought to exhibit a non-uniform distribution within tumor tissues, rather than a structured spatial distribution (14). The expression of TAM subtypes in the tumor core versus the peritumoral region also influences tumor dynamics and prognosis for patients (15, 16). Multi-regional sampling has proven instrumental in characterizing tumor heterogeneity, which identified the spatiotemporal evolutionary patterns within the TME by aggregating cellular components from multiple regions of the patients (17). In addition, the development of spatial transcriptomics (ST) technology has facilitated detailed examinations of distinct transcriptional profiles and cellular interactions across different spatial domains (18). Therefore, further characterization of the spatial heterogeneity of TAMs subsets is essential for comprehensive understanding the tumor ecosystem in HCC.

Reprogramming the polarization state is one important strategy in therapies targeting TAMs (19). TAMs are highly sensitive to their surroundings, and can be polarized into tumoricidal M1 and tumor-supportive M2 types (20). M1 TAMs contribute to the elimination of tumor cells and defense against pathogens, and M2 TAMs are primarily linked to facilitating tumor growth and suppressing immune responses (21, 22). However, some TAMs subsets may exhibit non-classical M1/M2 phenotypes or express M1 and M2 signatures non-exclusively, which epitomizing a narrower interpretation of TAMs (23, 24). Importantly, pioneering research in other solid tumors has highlighted the mutually exclusive expression of CXCL9/SPP1, rather than traditional M1/M2-signatures in TAMs (25, 26). The CXCL9:SPP1 (CS) expression ratio, or CS polarity, has been identified as a key determinant of whether TAMs adopt an anti-tumor or pro-tumor phenotype. However, the scenary of CS polarity in HCC and the underlying regulatory mechanisms warrant further exploration. Additionally, the potential of CS polarity as a prognostic marker has been highlighted, prompting further development toward clinical application (25, 27, 28). Radiomics, as a non-invasive approach, has shown promise in prognostic stratification of HCC by transforming routinely acquired medical images into higher-dimensional radiomics features and constructing predictive models (29). Therefore, developing a non-invasive imaging biomarker that reflects CS polarity is essential to facilitate its clinical translation.

In this study, we integrated multi-omics data to explore the heterogeneity of TAMs in HCC tumors. By employing multiple computational strategies, we specifically focused on the subpopulation characterized by elevated expression of SPP1 and CXCL9. SPP1^+^ TAM, located within the tumor core, cooperated with cancer-associated fibroblasts to induce extracellular matrix formation, thereby promoting HCC tumorigenesis. In contrast, CXCL9^+^ TAM, positioned in peritumoral regions, recruited CD8^+^ T cells to create an immunostimulatory micro-environment. We preliminarily identified the CXCL9:SPP1 polarization paradigm in HCC and revealed underlying regulatory mechanisms and associations with other TME components. Additionally, the CS polarity may serve as a reliable prognostic and micro-environmental status indicator for HCC patients. We developed a radiomics model, RCSP (Radiogenomics-based CXCL9/SPP1 Polarity), based on medical imaging data, to assist in non-invasively predicting the levels of this biomarker. In conclusion, our work unveils the applicability of the CXCL9/SPP1 polarization state in HCC for the first time and emphasizes the broad prospects for personalized treatment based on the CS polarity.

## 2 Materials and methods

### 2.1 Study design

**Figure 1:**
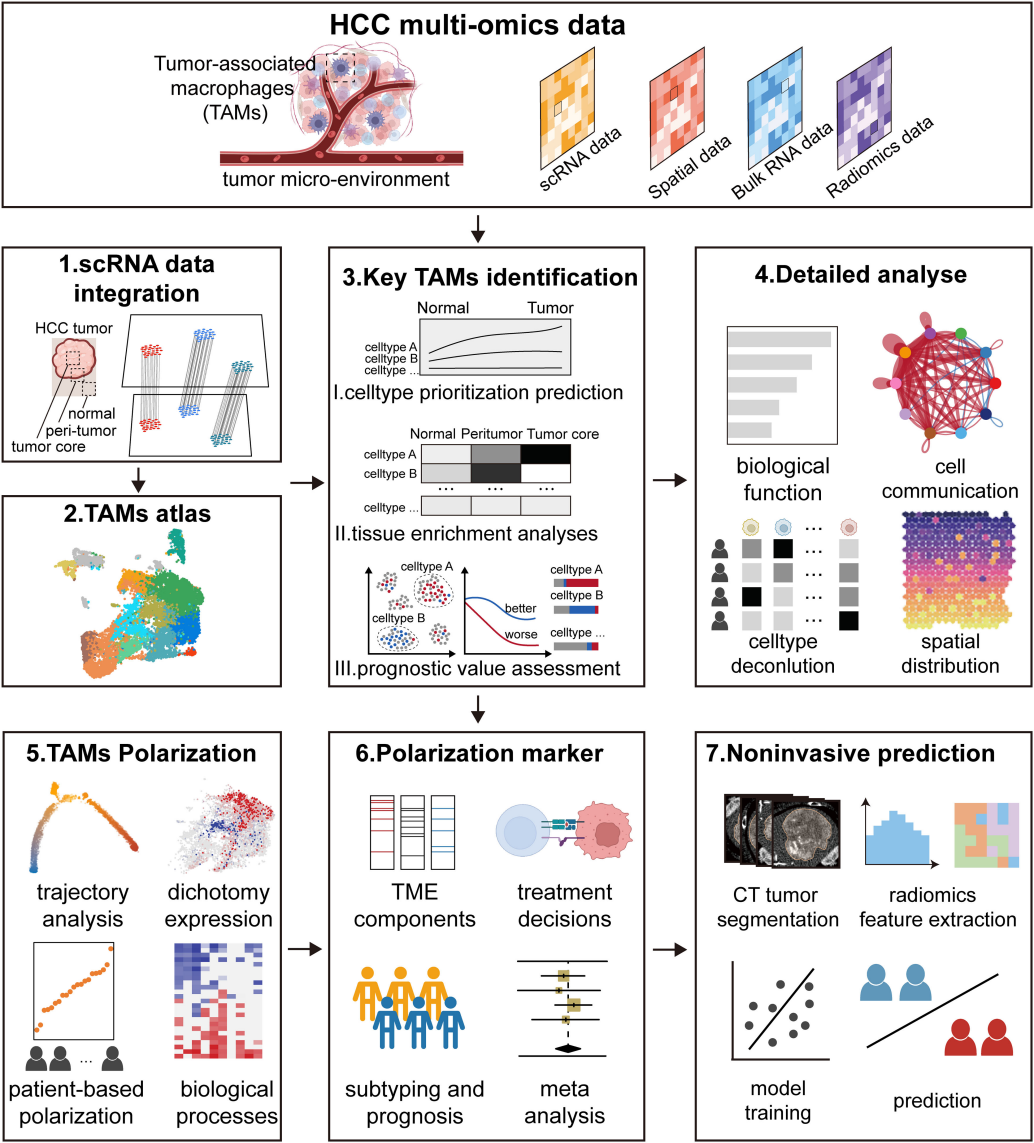
Study design. The study was designed to investigate the detailed landscape of tumor-associated macrophages (TAMs) within the hepatocellular carcinoma (HCC) micro-environment using multi-omics data, including single-cell data, spatial data, bulk data, and radiomics data. The study includes the following key aspects. Firstly, we integrated single-cell data from paired normal, peritumor and core tumor regions to construct an atlas of TAMs. Subsequently, we employed three distinct computational methods to identify the key subtypes of TAMs, followed by a detailed analysis combining multi-omics data. The study then focused on TAMs polarization based on trajectory analysis, dichotomy expression, patient-based polarization, and biological processes. In parallel, we developed markers reflecting TAMs polarization and explored the relationship between these markers and micro-environment components, treatment decisions, molecular subtyping, and prognosis. Finally, leveraging CT images from multiple cohorts, we developed a non-invasive predictive model to assess the polarization of TAMs within patients.

The study aimed to investigate the heterogeneous of TAMs within the HCC microenvironment using multi-omics data, including single-cell sequencing, spatial transcriptomics, bulk transcriptomics, and radiomics, as summarized in **Supplementary Table 1**. The study includes the following key aspects (**Figure 1**). Firstly, we integrated single-cell data from paired normal, peritumor and core tumor regions to construct an atlas of TAMs. Subsequently, we employed three distinct computational methods to identify the predominant subtypes of TAMs, followed by a detailed analysis combining multi-omics data. The study then focuses on TAMs polarization based on trajectory analysis, dichotomy expression, patient-based polarization, and biological processes. In parallel, we developed markers reflecting TAMs polarization and explored the relationship between these markers and micro-environment components, treatment decisions, molecular subtyping, and prognosis. Finally, leveraging CT images from multiple cohorts, we developed a non-invasive predictive model to assess the polarization of TAMs within patients.

### 2.2 Data collection and power analysis

Multi-regional scRNA-seq data of HCC were obtained from the Gene Expression Omnibus (GEO, https://www.ncbi.nlm.nih.gov/geo/) under accession codes GSE156337 (n=14), GSE140228 (n=6) and GSE189903 (n=4), encompassing a total of 24 patients and 167,169 cells with corresponding sampling locations, including normal tissue, peritumoral area, and the tumor core. These datasets were all performed using the 10×Chromium single cell platform (10×Genomics). To mitigate batch effects across these datasets, we integrated the samples using default parameters in the FindIntegrationAnchors and IntegrateData functions of Seurat package. In addition, we included a single-cell dataset from patients who underwent immunotherapy (n=20), consisting of 10 responders and 10 non-responders to treatment, to validate the results of spatial transcriptomics (https://lambrechtslab.sites.vib.be/en/aHCC). The batch correction of this dataset was processed was performed on a sample-by-sample basis.

Spatial transcriptomics data from tumor sections of responders (n=3) and non-responders (n=4), as well as normal sections of non-responders (n=3), were extracted from HCC patients receiving immunotherapy on Mendeley Data (identifier: skrx2fz79n) (30).

Bulk transcriptomics data from four cohorts of HCC patients (n=942) were included, which comprised the HCC cohort (TCGA-LIHC, n=425) from the TCGA data portal (http://gdac.broadinstitute.org/) and the LIRI-JP project cohort (n=232) from the International Cancer Genome Consortium (ICGC) portal (https://dcc.icgc.org/). The raw counts were transformed into TPM values. Additionally, two independent expression datasets based on microarray from GEO were included: GSE14520 (n=221) and GSE116174 (n=64). The clinical information is summarized in the **Supplementary Table 1**.

Two radiomics cohorts with clinical information and corresponding preoperative CE-CT imaging data of HCC patients were retrieved from the publicly accessible Cancer Imaging Archive (TCIA) database (https://www.cancerimagingarchive.net/). We excluded patients with missing images in the enhanced phase and those without reference tumor locations. Finally, we included the TCIA-TCGA-LIHC cohort (n=41), which could be matched with relevant transcriptomic information from the TCGA-LIHC, and the TCIA-HCC-TACE-Seg cohort (n=65), which consisted of HCC patients who underwent TACE pre-treatment contrast-enhanced CT scanning.

Power analysis was conducted using the SCOPIT website for single cell data, to determine the minimum number of cells required per sample to achieve 95% statistical power (31). Using the retrospective analysis mode, we first performed marker identification and clustering to determine the percentage of each cell type. Based on these results, we calculated the minimum number of cells needed to capture at least 10 cells from the lowest frequency cluster (representing 1% of the population) with 95% probability. This approach allowed us to estimate the required cell count for each sample to ensure sufficient power for downstream analyses. For the bulk and radiogenomics data, sample size estimation and power analysis were performed using the ssizeRNA package (v1.3.2), confirming that the sample size was sufficiently powered (power = 0.8, false discovery rate = 0.05) to detect differential expression between patients with high and low CXCL9/SPP1 polarity (32).

### 2.3 Quality control and preprocessing of scRNA-seq data

According to the standardized pipeline, the scRNA-seq data were processed using the Seurat package (version 4.3.0) in R software (33). Cells were meticulously screened as follows: (1) those containing fewer than 200 genes, or more than 5,000 genes were filtered out; (2) cells with a mitochondrial gene proportion exceeding 20%, as determined by the PercentageFeatureSet function, were excluded; (3) genes expressed in three or fewer cells were subsequently removed. After quality control, the gene expression matrices were normalized using the NormalizeData function, and (4) doublets were detected and removed using DoubletFinder (34).

After batch effect elimination, the NormalizeData function was used to normalize the expression matrix, and the FindVariableFeatures function was applied to identify the top 2,000 highly variable genes (HVGs). For dimensionality reduction and clustering on the expression matrix, ScaleData was used to scale the data, and RunPCA was performed to analyze the first 50 principal components. The results were then clustered using FindNeighbors (resolution=0.6) and FindClusters and projected onto two-dimensional uniform manifold approximation and projection (UMAP) embedding images for visualization. The characteristic genes of each cell type were identified using the FindAllMarkers function, with a logFC threshold of 0.25 and a minimum fraction of genes detected in cells set at 0.1.

### 2.4 Annotation of cell clusters

Based on a previous study (35), the CellMarker 2.0 and PanglaoDB databases (36, 37), the expression of specific features was considered the main basis for cluster annotation. We performed a two-round annotation process.

First, hepatocytes (expressing HP and KRT8), endothelial cells (expressing ACTA2 and RGS5), fibroblasts (expressing GNG11 and VWF), and immune cells (expressing PTPRC) were distinguished. Immune cells were further categorized into macrophages (expressing CD163 and CD68), monocytes (expressing S100A8 and S100A9), dendritic cells (CD1E and CD1C), mast cells (expressing TPSB2 and KIT), natural killer cells (expressing NKG7 and KLRF1), B cells (expressing CD79A and MZB1), and T cells (expressing CD3D and CD3E). Then, macrophages were re-integrated and further categorized into different subtypes based on characteristic genes and marker genes obtained in a previous study (35). The biological functions of these subtypes were analyzed through enrichment analysis using the GO databases with the clusterProfiler package (version 4.9.2).

### 2.5 Identification of predominant macrophage subtype in HCC

We employed three computational methods to comprehensively evaluate the predominant macrophage subtype in HCC tumorigenesis. (1) Cell-type prioritization analysis. The Augur algorithm was used to identify which cell types are most responsive to biological perturbations (38). This method utilizes a random forest classifier on subsampled matrices and reports the mean cross-validation area under the receiver operating characteristic curve (AUC) across these subsamples. An AUC value of 0.5 suggests that there is no significant difference in perturbation between cells from the tumor site and those from the normal state within a cluster. In contrast, an AUC value of 1.0 indicates that cells from the tumor site exhibit a higher degree of perturbation compared to the normal state. (2) Tissue enrichment analysis (39). To quantify the enrichment preference of cell types across different tissue groups, we compared the observed and expected cell numbers for each cluster within each tissue group using the following formula: Ro/e = (Observed/Expected). The expected cell numbers for each cell type in the tissues were derived from the Chi-squared test. We considered a cluster to be enriched in a specific tissue if the Ro/e ratio was greater than 2. (3) Prognostic assessment (40). The Scissor algorithm was applied to identify cell types associated with the survival outcomes of HCC patients in the TCGA-LIHC cohort. Overall survival (assessed using Cox regression) served as the dependent variable. The alpha parameter was set at 0.01, and the cutoff for the percentage of Scissor-selected cells among the total cells was set at 0.2. The reliability significance test was conducted using the reliability.test function.

### 2.6 Cell communication analysis

Cell communication analysis was performed using the CellChat package (version 2.0.0) to explore potential interactions between cell types (41). First, we constructed a CellChat object using scRNA-seq data from HCC and normal liver tissues. We then utilized the CellChat database to investigate cell-cell communication and identify ligand-receptor pairs within these cell populations. These interactions were further analyzed to identify specific communication patterns. The probability of communication between cells was computed to examine the molecular interaction networks between various cell types, with particular focus on the communication between TAM subtypes, especially SPP1^+^ TAMs and CAFs. Additionally, to assess the relationship between CXCL9^+^ TAMs and immunotherapy response, we constructed a separate CellChat object using scRNA-seq data from immunotherapy responders and non-responders. This analysis focused on the differential communication patterns of CXCL9^+^ TAMs in CXCL signaling pathways between T cell subpopulations.

### 2.7 Trajectory analysis

To investigate the single-cell trajectories of TAM subtypes and the cell state transitions between CXCL9^+^ and SPP1^+^ TAMs, we performed trajectory analysis in DDR-Tree method with default settings using the R package Monocle2 (version 2.29.0) (42). Differentially expressed genes were identified for each cluster using the differentialGeneTest function, and cells were ordered in pseudotime based on genes with a q-value less than 0.05. We focused on the differences in the trajectories of CXCL9^+^ and SPP1^+^ TAMs across distinct trajectory branches, with the pattern of each cell on the branches determined by the expression levels of markers. Following trajectory construction, we again used the differentialGeneTest function to identify DEGs along the pseudotime continuum. This analysis was complemented with transcription factor activity analysis to identify potential therapeutic targets driving the phenotypic changes in CXCL9^+^ and SPP1^+^ TAMs.

### 2.8 Transcription factor activity analyses

DoRothEA analysis was used to infer the key transcription factors (TFs) of CXCL9^+^ and SPP1^+^ TAMs. DoRothEA (version 1.14.1) is a gene set resource that includes tTFs and their interactions with target genes, which enables the inference of TF activity from gene expression data (43). The TF activity scores, represented by the viper score, were estimated for cell clusters using DoRothEA, with the analysis conducted within a database that contains interactions with a specified confidence level. Subsequently, the identified TFs were combined with the DEGs of trajectory analysis to identify potential therapeutic targets driving the phenotypic changes of CXCL9^+^ and SPP1^+^ TAMs.

### 2.9 Estimation of cell type infiltration from bulk transcriptome data

The CIBERSORTx deconvolution algorithm (https://cibersortx.stanford.edu/) was employed to quantitatively estimate the subtypes of TAMs and the infiltration levels of CAFs in the TCGA-LIHC cohort (44), using absolute mode. The integrated scRNA-seq data from this study served as the reference for constructing the signature matrix. When creating the signature matrices, CIBERSORTx was executed with quartile normalization disabled for RNA-seq datasets. The permutation parameter was set to 500 iterations, and all other parameters were maintained at their default settings. To assess the relationships among the proportions of cell type infiltration, Pearson’s correlation analysis was conducted. Correlations were considered significant if the absolute value of the correlation coefficient (|R|) exceeded 0.3 and the false discovery rate (FDR) was less than 0.05.

### 2.10 Spatial transcriptomics data processing and analysis

Spatial transcriptomics data processing and visualization were conducted using the Seurat package (version 4.3.0). For normalization of the ST data, we employed the SCT method. The functions SelectIntegrationFeatures, PrepSCTIntegration, FindIntegrationAnchors, and IntegrateData were sequentially applied to integrate the ST data. To delineate the tumor area, we utilized the BoundaryDefine function from the Cottrazm R package (version 0.1.1) (45). The cellular composition of each spot was deconvoluted using the SpatialDecon function. To visualize the spatial co-localization of CAFs, SPP1^+^ TAMs, CXCL9^+^ TAMs, and CD8^+^ T cells in ST slices, we scored the top 20 DEGs of cell clusters with the AddModuleScore function. The SpatialDimPlot and SpatialFeaturePlot functions were combined to visualize the cell expression levels within the ST data.

PROGENy (version 1.24.0) was utilized to estimate the activity of 14 oncogenic pathways for each spot (46). The model calculates pathway activity by considering the expression levels of genes that are more responsive to perturbations within those pathways. NicheNet (version 2.0.4) was employed to infer the mechanisms of interaction between SPP1^+^ TAMs and CAFs with malignant cells in tumors (47). Specifically, we used the GetTissueCoordinates function from the Seurat package to obtain the spatial coordinates of the spots across different HCC samples. We then computed the Euclidean distance between each pair of spots. Hepatocyte or tumor cell spots were selected in normal or tumor sections, respectively. Subsequently, fibroblast or SPP1^+^ TAMs-CAFs spots were chosen, ensuring that these spots were within a distance no greater than 2.5 times that of the hepatocyte or tumor cell spots. Malignant cells were designated as receiver cells, and hepatocytes served as reference cells. The ligand_activity_target_heatmap function was applied to visualize the regulatory activity of ligands.

### 2.11 Multiplex immunofluorescence staining

Multiplex immunofluorescence (mIF) staining was performed on liver tissue sections obtained from HCC mouse model. The animal experimental protocols were approved by the Institutional Animal Care and Use Committee of Nanjing University of Chinese Medicine (approval number: 202312A034). Tissue sections were incubated 10 minutes with citrate buffer (10 mM) at 98°C for antigen retrieval, and blocked 1 hour with 5% bovine serum albumin (BSA) at room temperature. Following this, tissue sections were incubated overnight at 4°C with the primary antibodies, which included anti-SPP1/Osteopontin (Proteintech, China), anti-CXCL9 (Invitrogen, USA), anti-CD68 (Boster, China), anti-α-SMA (Boster, China), and anti-CD8 (Abcam, UK). The sections were then incubated with the corresponding secondary antibodies: Alexa Fluor 488-conjugated goat anti-rabbit (Abcam, UK), Alexa Fluor 594-conjugated goat anti-rabbit (Abcam, UK), and Cy3-conjugated goat anti-rabbit (Abcam, UK) for 1 hour at room temperature. Finally, the sections were mounted with DAPI-containing antifade medium (Sigma-Aldrich, USA), and images were captured using a fluorescence microscope (Nikon, Japan). The results are expressed as cell density (cells/mm^2^), calculated by dividing the total number of positive cells by the total area.

### 2.12 Individual-based method for CS polarity definition

To further understand the role of CXCL9:SPP1 TAM polarity (CS polarity) in the TME, we implemented an individual-based method (25) for determining the relationship between CS polarity and the gene expressions and biological function in diverse cell types. For scRNA-seq data, the CS polarity was defined as the ratio of the adjusted mean counts of gene CXCL9 to those of gene SPP1 in TAMs. We first established the adjusted mean counts, the calculation of the adjusted mean count for gene *G* in cell type *C* for patient *P* is as follows:

1. Compute the mean expression. For gene *G* in cell type *C* of patient *P*,


$${\bar{X}}_{P,C,G}=\frac{1}{m}\sum_{i=1}^{m}X_{P,C,G}(i)$$

where *m* is the cell numbers of cell type *C*, and $X_{P,C,G}(i)$ is the expression value of gene *G* in cell *i*.

2. Calculate the scaling factor. First, we calculate the average expression ${\bar{X}}_{P,C}$ for all genes in cell type *C* for patient *P*, and then the average expression ${\bar{X}}_{C}$ for all genes in cell type *C* across all patients *N*.


$${\bar{X}}_{P,C}=\frac{1}{g}\sum_{j=1}^{g}{\bar{X}}_{P,C,j}$$

$${\bar{X}}_{C}=\frac{1}{N}\sum_{k=1}^{N}{\bar{X}}_{k,C}$$

The scaling factor $S_{C}$ is finally calculated as follows,


$$S_{C}=\frac{{\bar{X}}_{P,C}}{{\bar{X}}_{C}}$$

where *g* is the total number of genes in cell type *C* for patient *P*, ${\bar{X}}_{P,C,j}$ is the average expression of the gene *j* in cell type *C* for patient *P*, and ${\bar{X}}_{k,C}$ is the average expression of all genes in cell type *C* for the patient *k*.

3. Calculate the adjusted mean counts. We multiply the average expression ${\bar{X}}_{P,C,G}$ obtained in step (1) by the scaling factor $S_{C}$ in step (2) to obtain the adjusted average count ${\bar{X}}_{P,C,G}^{adj}$ .


$${\bar{X}}_{P,C,G}^{adj}={\bar{X}}_{P,C,G}\times S_{C}$$

After the adjusted mean counts established, the CS polarity for patient *P* is defined as follows,


$$CS\_Ratio=\frac{{\bar{X}}_{P,TAM,CXCL9}^{adj}}{{\bar{X}}_{P,TAM,SPP1}^{adj}}$$

where ${\bar{X}}_{P,TAM,CXCL9}^{adj}$ is the adjusted mean counts of CXCL9 in TAMs for patient *P*, and ${\bar{X}}_{P,TAM,SPP1}^{adj}$ is the adjusted mean counts of SPP1.

### 2.13 Radiomics feature extraction and RCSP model construction

Two radiomics cohorts from the TCIA database, TCIA-TCGA-LIHC (n=41) and TCIA-HCC-TACE-Seg (n=65), comprising a total of 106 HCC patients with contrast-enhanced CT (CE-CT) images, underwent radiomics analysis. Patients in the TCIA-TCGA-LIHC cohort served as the training set due to the availability of matched transcriptomic information. The TCIA-HCC-TACE-Seg cohort patients were utilized as the validation set. Tumor reference coordinates were retrieved from the TCIA website (48). For the volume of interest (VOI) of the tumor, lesions were manually annotated on both arterial and portal venous phase images, using reference markups and segmented with a threshold-based segmentation algorithm via 3D Slicer software. The area beyond the liver parenchyma was excluded, along with large vessels, adjacent organs, and air cavities. Image preprocessing and feature extraction were conducted using the PyRadiomics tool (version 3.0.1) in Python (49). Images were resampled to a voxel size of 1 × 1 × 1 mm³ to standardize voxel spacing and discretized with a fixed bin width of 25.

A two-stage modeling strategy was implemented to develop the RCSP (Radiogenomics-based CXCL9/SPP1 Polarity) model. Stage 1: Radiomics score (RadScore) construction. The radiomics features extracted from each VOI were categorized into three classes: (1) first-order features (n=18), (2) shape features (n=14), and (3) texture features (n=68). Features were calculated on both the original image and the filtered image. In the training set, radiomics features significantly correlated with the CS polarity (|r| > 0.3, P < 0.05) were initially identified using Pearson correlation after Z-score normalization. Feature selection was then performed using the least absolute shrinkage and selection operator (LASSO) regression model, following the determination of the optimal λ value through leave-one-out cross-validation. The RadScore for each sample was calculated based on the coefficients of the selected features in the model. Subsequently, this cohort was subjected to correlation analysis, receiver operating characteristic (ROC) curve analysis to predict CS polaritys in patients. Stage 2: Clinical indicators incorporation. To enhance prognostic robustness, we combined the RadScore with clinical indicators. The clinical indicators common across both training and validation cohort were selected for inclusion, and assessed via univariate Cox proportional hazards regression (P < 0.15). The filtered variable were combined with the precomputed RadScore into the Cox proportional hazards regression models, and the risk score for each patient was calculated using the following formula:


$$\text{RiskScore }=\;{\hat{\beta }}_{RadScore}\cdot Radscore+\sum {\hat{\beta }}_{j}\cdot X_{j}^{\left(clinical\right)}$$

Where ${\hat{\beta }}_{RadScore}$ is the estimated coefficient for the RadScore derived from the model, $X_{j}^{\left(clinical\right)}$ represents the clinical covariates, and ${\hat{\beta }}_{j}$ are the estimated coefficients for each clinical indicator $X_{j}^{\left(clinical\right)}$ .

Patients in the training cohort were stratified into high-risk and low-risk subgroups using the median RiskScore as threshold. Survival differences between subgroups were evaluated using Kaplan-Meier analysis with log-rank testing. The model with fixed coefficients was applied to the validation cohort. The patient stratification was stratified using the predefined threshold of risk score from the training cohort, and survival curves were compared following the same protocol.

### 2.14 Statistical analysis

Statistical and bioinformatics analyses, as well as data visualization and plotting, were conducted using R software (version 4.3.0) and Python (version 3.7). The nonparametric Wilcoxon test was employed to investigate differences in continuous and categorical variables between the two groups. For comparisons among three or more groups, the Kruskal-Wallis test and one-way ANOVA were applied. The Benjamini-Hochberg method was utilized to adjust the P-values for multiple testing, implemented via the R function p.adjust. Proportions were compared using the chi-squared test or Fisher’s exact test, as appropriate. Survival analysis was conducted using the Kaplan-Meier method, facilitated by the R package survival (version 3.5.5). Independent prognostic analysis was performed using univariate and multivariate Cox proportional hazard regression models, which estimated the hazard ratio (HR) and the 95% confidence interval (CI) concurrently. The meta-analysis and the generation of forest plots were completed using the R package meta (version 6.5.0). A P-value of less than 0.05 was considered to indicate statistical significance.

## 3 Results

### 3.1 Construction of multi-regional TAMs atlas in HCC

**Figure 2:**
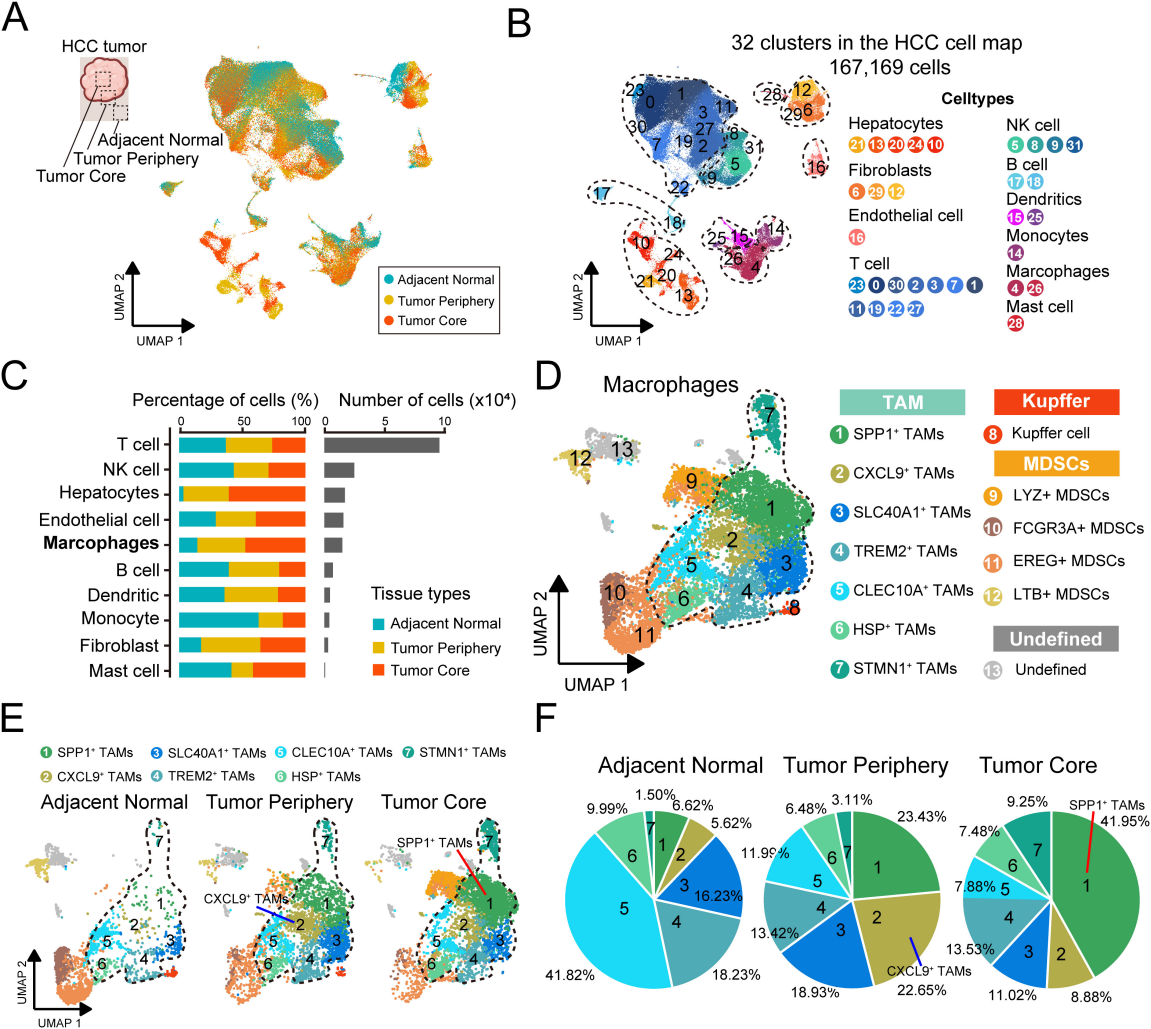
Construction of the TAMs transcriptional atlas across multi-region samples. **(A)** Datasets with multi-regional samples were merged in this study, batch effect was corrected and projected onto the bidimensional UMAP space. **(B)** UMAP shows the distribution of major cell types, colored by subtypes. **(C)** The proportion of major cell types showing in bar plots in different tissues (left), and total cell number of each cell type (right) are shown. **(D)** UMAP visualization of macrophages subtypes, colored by subtypes. **(E)** UMAP visualization of macrophages subtypes across sampling regions. **(F)** Pie charts showing the proportion of cells per tissue in each TAM clusters.

To systematically characterize macrophages in HCC progression, we integrated three scRNA-seq datasets from Zhang et al. (n = 7), Sharma et al. (n = 8), and Wang et al. (n= 6) containing 173,614 cells from different sampling locations of 21 primary HCC tumors. After preprocessing and batch effects mitigation, 167,169 cells were kept and grouped into transcriptionally distinct clusters using graph-based clustering (**Figure 2A**, **Supplementary Figures 1A, B**). In total, 32 distinct clusters were identified, and we annotated each cluster with its respective markers. Cells were classified into 10 major cell types (**Figure 2B**, **Supplementary Figure 1C**), including hepatocytes (n=13,269) identified by the expression of HP and KRT8, fibroblast (n=2,813) which were positive for GNG11 and VWF expression, endothelial cells (n=12,092) marked by ACTA2 and RGS5, T cells (n=96,086) which expressed the T-cell receptor signaling mediators CD3D and CD3E, NK cell (n=19,501) identified by NKG7 and KLRF1 expression, B cells (n=5,147) marked by CD79A and MZB1, dendritic cell (n=3,856) marked by CD1E and CD1C, monocyte (n=3,569) defined by their classical markers S100A8 and S100A9, macrophage (n=10,518) which were positive for CD163 and CD68 expression, and mast cells (n=318) marked by TPSB2 and KIT. Using SCOPIT power analysis, we showed that the cell types with the lowest frequency in each sample can be detected in other samples with high confidence of 95% using the number of cells captured (**Supplementary Figures 2A, B**).

We analyzed cellular distribution across different sampling sites and found that T cells and NK cells were the most abundant (**Figure 2C**). Notably, the proportion of hepatocyte infiltration in the adjacent normal tissue was remarkably low, likely due to fibrosis, extracellular matrix remodeling, and chronic inflammation in the surrounding tissue, which may have contributed to hepatocyte degeneration. This observation was further corroborated by an additional HCC study (50) (**Figure 2C**, **Supplementary Figure 1D**). We focused on the macrophages. Compared to normal tissues, the infiltration of macrophages was increased in tumor tissue (**Figure 2C**). We re-clustered the macrophage population and employed specific cellular signature markers reported before performing an in-depth annotation (**Figure 2D**, **Supplementary Figure 1E**). The subsets were classified into three broad categories, (1) monocyte-derived macrophages (TAMs, cluster 1-7), (2) resident liver macrophages (Kupffer cells, cluster 8), and (3) myeloid-derived suppressor cell precursors (MDSCs, cluster 9-12). Resident liver macrophages, also known as Kupffer cells, were marked by high expression of the classic marker MARCO, which played a vital component in the innate immune system (51). MDSCs are a heterogeneous population of myeloid cells that are derived from the bone marrow, characterized by high expression of FCN1 (52).

TAMs consisted of seven subclusters with dominant subset-specific genes, respectively, including SPP1^+^, CXCL9^+^, SLC40A1^+^, TREM2^+^, CLEC10A^+^, HSP^+^ and STMN1^+^ TAMs (**Figure 2D**). Representative genes of each subclusters were summarized (**Supplementary Table 2**). SPP1^+^ TAMs showed high levels of secreted phosphoprotein 1 (SPP1) expression, a key component of the epithelial-mesenchymal transition pathway, and were involved in extracellular matrix receptor communication (53). CXCL9^+^ TAMs expressed a variety of genes within the chemokine family (e.g. CXCL9, CXCL10) associated with positive responses to immune checkpoint blockade in antitumor immunity (54). SLC40A1^+^ TAMs expressed ferroprotein SLC40A1, the only known cellular iron exporter in mammals (55). In TREM2^+^ TAMs, gene expressions were characterized that are primarily involved in the transmembrane receptor of the immunoglobulin superfamily, which is widely implicated in immunoinflammatory responses seen in the pathobiology of several diseases (56). CLEC10A^+^ TAMs expressed high levels of the CLEC10A gene, which could recognize and act on tumor-associated antigens and effectively present the antigens to T cells (57). HSP^+^ TAMs expressed several heat shock protein genes associated with cellular heat response and angiogenesis regulation (58). And STMN1^+^ TAMs exhibited high expression of genes associated with cell proliferation (59).

We compared the degree of infiltration for each TAMs subtype, revealing an uneven distribution of relative abundance (**Figures 2E, F**). We observed that SPP1^+^ TAMs (tumor core: 41.95%, peri-tumor: 23.43%), CXCL9^+^ TAMs (8.88%, 22.65%), TREM2^+^ TAMs (13.53%, 13.42%) and SLC40A1^+^ TAMs (11.02%, 18.93%) mainly existed in tumor region, while the other subtypes, like CLEC10A^+^ TAMs (normal: 41.82%) were notably present in normal tissues, which may reflect the functional heterogeneity of the subsets in the TME.

### 3.2 SPP1^+^ TAMs represent a predominant subset associated with poor prognosis

To identify the specific TAMs subsets that significantly influence HCC progression, we performed three methods to comprehensively evaluate the predominant macrophage subtype in HCC tumorigenesis, including (1) cell-type prioritization, (2) tissue enrichment analyses and (3) prognostic assessment. The strategy assumed that if a specific cell type exerts a significant influence on tumor progression, it would be responsive to biological perturbations during the disease and exert function by migrating toward the tumor cells, thereby influencing the prognosis in patients.

**Figure 3:**
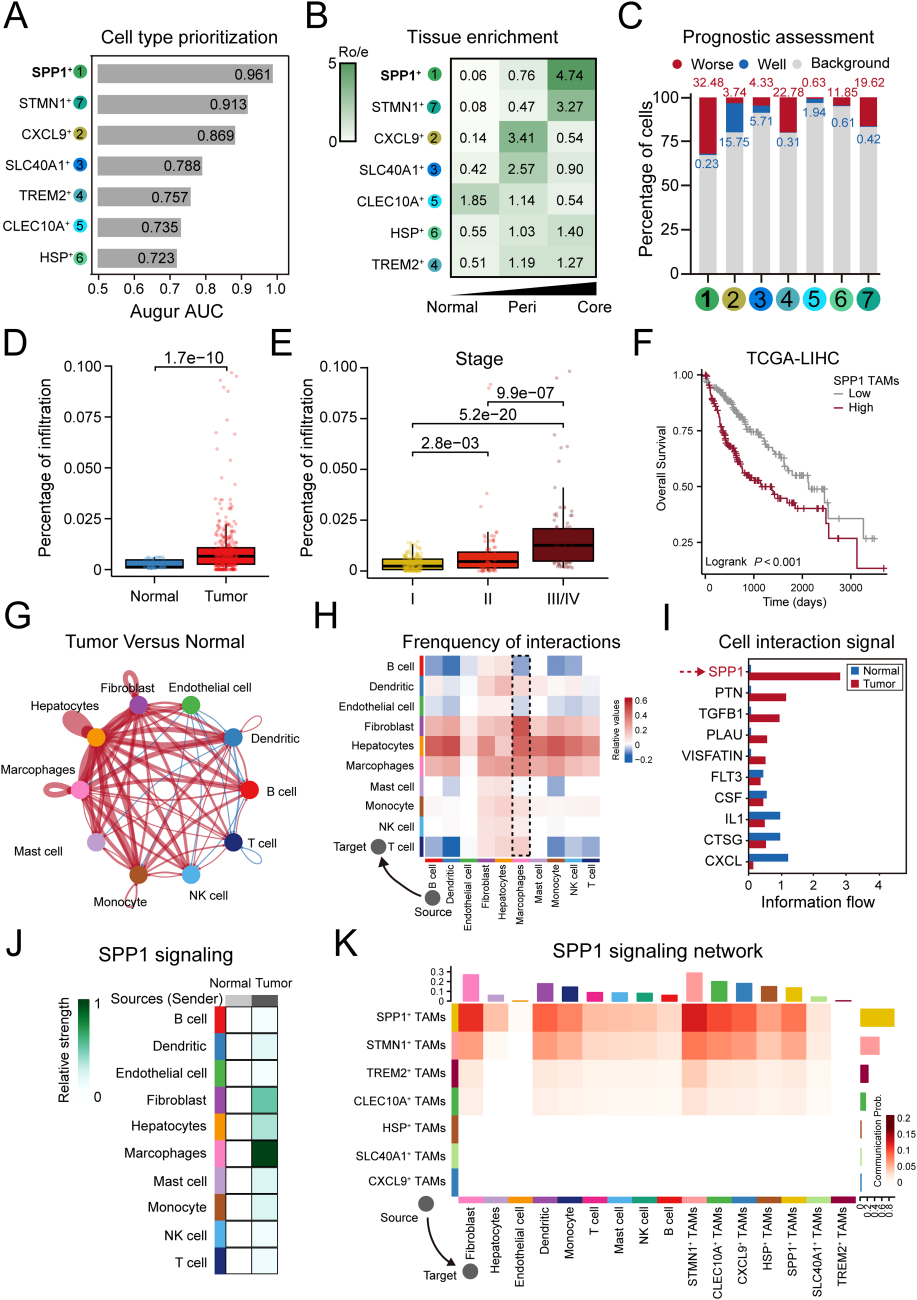
SPP1^+^ TAMs represent a predominant worse prognosis subset in HCC tumorigenesis. **(A)** Bar plot displaying the AUC of cell type prioritizations calculated by Augur. **(B)** Heatmaps showing the R_Observed/Expected_ (Ro/e) of TAM subtypes occurring in each tissue. **(C)** Bar plot showing the visualization of Scissor analysis of TAMs subtypes. The cellular proportion of cells related to poor or good prognosis in each TAMs subtype. **(D)** Significant higher ratio of SPP1^+^ TAMs in tumor than normal tissue in TCGA-LIHC cohort. **(E)** SPP1^+^ TAMs infiltration is upregulated as tumor stage increases. **(F)** Higher SPP1 TAMs infiltration was associated with worse overall survival. **(G, H)** Circle plots **(G)** and heatmap **(H)** showing the change in cell communication intensity between major cell types in HCC tumor compared to normal tissues. Lines and squares in red indicating increased cell communication in HCC tumors, and in blue decreased. **(I)** Significant signaling pathways ranked based on differences in the overall information flow within the inferred networks between the tumor and normal groups. The red bar indicated top pathways enriched in tumor group, and blue indicated enriched in normal group. **(J)** The inferred SPP1 signaling networks between major cell types in tumor compared to normal tissue. Different shades of colors indicated the communication probability. **(K)** The inferred SPP1 signaling networks between TAM subtypes and other cell types. The darker the color the more communication probability is inferred in tumors. The top-colored bar plot represents the sum of column of values displayed in the heatmap (incoming signaling). The right-colored bar plot represents the sum of row of values (outgoing signaling).

Firstly, to evaluate the responses of TAMs to tumorigenesis, we performed Augur analyses to prioritize the perturbation of the TAMs subtypes. We found that SPP1^+^ TAMs exhibited the most profound change, with the highest AUC of 0.961. The STMN1^+^ TAMs (AUC=0.913) and CXCL9^+^ TAMs (AUC=0.869) also responded to tumorigenesis, but to a lower extent (**Figure 3A**). Secondly, we performed a χ^2^ test comparing the observed and expected cell numbers in each cluster (R_O/E_). Specially, SPP1^+^ TAMs and STMN1^+^ TAMs were enriched in tumor core region, and SPP1^+^ TAMs showed the highest R_O/E_ value of 4.74. The CXCL9^+^ TAMs and SLC40A1^+^ TAMs were enriched in peri-tumor region, and CXCL9^+^ TAMs showed the highest R_O/E_ value of 3.41 (**Figure 3B**). Lastly, the Scissors analysis was performed to identify TAMs subtypes associated with the survival outcomes from HCC patients. We identified 1,351 Scissor^+^ TAMs that were associated with worse survival and 215 Scissor^−^ TAMs that were associated with better survival (**Figure 3C**, **Supplementary Figure 3A**). Notably, SPP1^+^ TAMs, TREM2^+^ TAMs and STMN1^+^ TAMs showed worse survival correlation, and SPP1^+^ TAMs accounted for the highest proportion among Scissor^+^ TAMs (32.48%). CXCL9^+^ TAMs, SLC40A1^+^ TAMs and CLEC10A^+^ TAMs showed better survival correlation, and CXCL9^+^ TAMs accounted for the highest proportion in Scissor^−^ TAMs (15.75%) (**Figure 3C**). To further investigate the relationship between the SPP1^+^ TAMs and tumor progression, we employed the deconvolution algorithm to infer the cell proportion for each sample from the bulk RNA-seq data. Our findings revealed that the infiltration of SPP1^+^ TAMs was markedly elevated in tumor tissues relative to normal tissues (**Figure 3D**), and the proportion of SPP1^+^ TAMs increased with the advancement of tumor stage (**Figure 3E**). Furthermore, a high abundance of SPP1^+^ TAMs was associated with a reduced overall survival rate for HCC patients (**Figure 3F**). Altogether, these results suggested that SPP1^+^ TAMs maybe a predominant subset associated with poor prognosis in HCC tumorigenesis.

The cell-cell interaction (CCI) networks among cell populations exert a profound impact on the development and metastasis of HCC (60). We compared the strength of inferred interactions and found that the CCI network of cell types in HCC exhibited a higher level of interaction strength compared to that of normal tissue (**Figure 3G**). We found that macrophages, which served as the source cells of tumor, showing stronger interaction strength with other cell types, especially fibroblasts and hepatocytes (**Figure 3H**), and the SPP1, PTN, CCL, TGFB1 and PLAU pathways were more enriched in tumor regions (**Figure 3I**). The SPP1 pathway was the most enriched pathway in tumor region, which has been reported to lead to immunosuppression in the TME by binding to its receptor CD44 (61). In the SPP1 signaling network, macrophages were the primary source for cell communication (**Figure 3J**), and compared to other TAMs subtypes, the strength of SPP1-mediated interactions sourcing from SPP1^+^ TAMs was markedly higher (**Figure 3K**). Taken together, these results demonstrate that the SPP1 signaling was a critical mediator of cell communication in HCC tumorigenesis and that SPP1^+^ TAMs may play an important role in this process.

### 3.3 SPP1^+^ TAMs and CAFs synergistically contribute to pro-tumorigenic micro-environment in HCC

**Figure 4:**
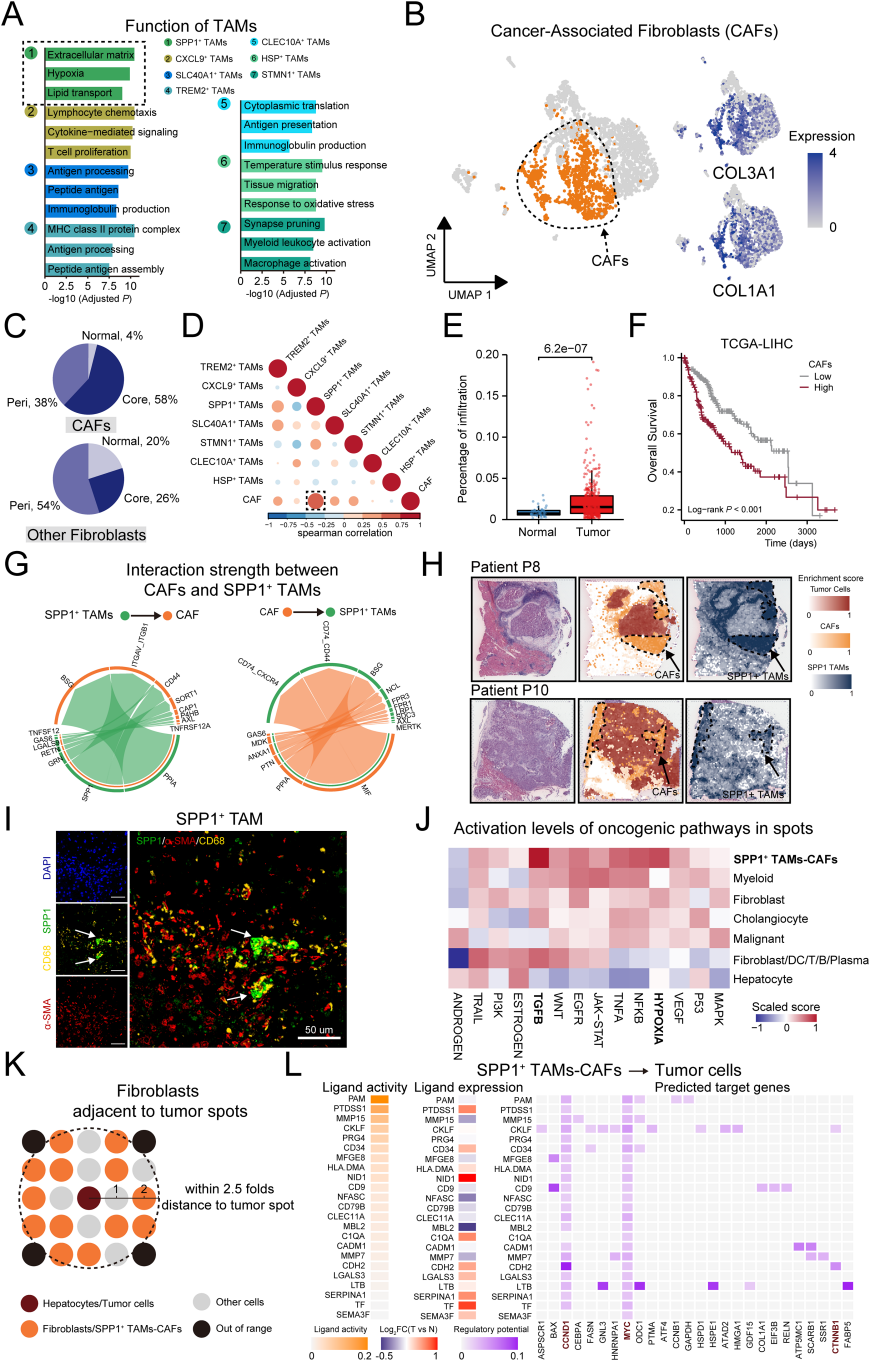
SPP1^+^ TAMs and CAFs synergistically contribute to pro-tumorigenic micro-environment in HCC. **(A)** Bar plot showing the GO enrichment analysis based on the top 50 significantly expressed genes of each TAMs subset. **(B)** Left: UMAP showing the distribution of fibroblasts, the CAFs were colored by orange. Right: Expression of canonical markers for CAFs. **(C)** Pie chart indicating the percentage of tissue distribution on CAFs and non-CAFs. **(D)** Heatmaps exhibit the correlation between Cancer-associated fibroblast and TAM subtypes in TCGA-LIHC cohort, the correlation between CAF and SPP1 TAM marked with a black dotted box. **(E)** Significantly higher ratio of CAFs in tumor than normal tissue in TCGA-LIHC cohort. **(F)** Higher CAF infiltration was associated with worse overall survival. **(G)** Circos plot shows cell communication from SPP1^+^ TAMs to CAFs, and from CAFs to SPP1^+^ TAMs. **(H)** Scores of the CAF and SPP1^+^ TAMs signatures enrichment in each spot on ST sections. **(I)** Representative mIF staining of liver tissues from the HCC mouse model. DAPI (blue), CD68 (yellow), SPP1 (green), α-SMA (red) are shown, along with individual and merged channels. Scale bar, 50 μm. **(J)** Activation levels of oncogenic pathways in defined cell types of each spot. **(K)** Schematic representation of the selection of fibroblasts/SPP1^+^ TAMs-CAFs spots near hepatocytes/malignant cells spots in normal and tumor tissue sections. **(L)** Left: Heatmap displaying the expression levels of ligands highly expressed in SPP1^+^ TAMs-CAFs. Right: the expression levels of corresponding target genes on tumor cells.

We further conducted functional enrichment analysis on TAMs subtypes (**Figure 4A**). Compared to other subtypes, SPP1^+^ TAMs were uniquely and significantly enriched in extracellular matrix (ECM). Recognizing the critical role of ECM in tumor progression through dynamic remodeling (62), and identifying cancer-associated fibroblasts (CAFs) as key cellular components in ECM remodeling (63, 64), we hypothesized that SPP1^+^ TAMs may synergize with CAFs to facilitate tumor progression.

To validate this assumption, we selected the CAFs in the fibroblast population, which was labeled by COL3A1 and COL1A1 (**Figure 4B**). We observed that CAFs mainly existed in tumor regions, especially in tumor core region (58%), which is consistent with the observation on SPP1^+^ TAMs (**Figure 4C**). Pathway analysis of differentially expressed genes revealed CAFs were related to ECM organization (**Supplementary Figure 3B**). We performed deconvolution analysis to assess the infiltration of TAMs and CAFs subsets in the TCGA-LIHC cohort. We calculated the pairwise correlations within the infiltrations of these subsets and found that the CAFs and SPP1^+^ TAMs were the most highly correlated populations in the examined cohort (**Figure 4D**). In addition, the infiltration proportion of CAFs was significantly increased in tumors compared to normal tissues, and patients with higher infiltration of CAFs had shorter overall survival (**Figures 4E, F**). Consistently, the interaction strength of cellular communications from SPP1^+^ TAMs to CAFs was markedly higher than that from other TAM subsets (**Supplementary Figure 3C**). The SPP1 signaling, by interacting with the relevant receptors (ITGAV, CD44) on CAFs, induced the formation of the ECM. Furthermore, the MIF/CD74 axis may represent the primary signaling pathway that promotes the infiltration of SPP1^+^ TAMs (**Figure 4G**).

We further examined whether CAFs and SPP1^+^ TAMs co-localized in HCC tissues. Using spatial transcriptomics data, the signature scores in CAFs and SPP1^+^ TAMs highlighted co-localization in the same spot (**Figure 4H**, **Supplementary Figure 4A**). In addition, the scores showed a significantly positive correlation (**Supplementary Figure 4B**). Multiplexed immunofluorescence (mIF) staining demonstrated that SPP1-positive and aSMA-positive cells were in close proximity in HCC slides (**Figure 4I**, **Supplementary Figure 4D**). We further investigated the synergistic effect of SPP1^+^ TAMs-CAFs on tumor cells. Based on the originally defined cell types in each spot from the previous research (30), we further compared the activation levels of oncogenic pathways, and found that the TGF-β and hypoxia pathways were more activated in SPP1^+^ TAMs-CAFs (**Figure 4J**). In addition, we explored the mediators and downstream targets of the SPP1^+^ TAMs/CAFs-tumor cell axis. In detail, we selected hepatocyte or tumor cell spots in normal or tumor sections, respectively. We then selected fibroblast or SPP1^+^ TAMs-CAFs spots, accordingly, ensuring that these spots were within a distance no greater than 2.5 times that of the hepatocyte or tumor cell spots (**Figure 4K**). We inferred the communication network between those spots. Interestingly, compared to normal tissues, SPP1^+^ TAMs-CAFs regulated tumor cells through the regulation of genes related to HCC tumorigenesis, including CCND1, MYC, CTNNB1, and BAX (**Figure 4L**). Moreover, these genes are involved in processes such as liver development, cell proliferation, and telomere organization, suggesting that SPP1^+^ TAMs-CAFs may participate in a synergistic action through these processes. This regulatory network could potentially enhance the malignant potential of tumor cells by influencing critical cellular functions and pathways. Collectively, these results suggest the pro-tumorigenic micro-environment may be regulated by the interaction of CAFs and SPP1^+^ TAMs.

### 3.4 CXCL9^+^ TAMs display distinct differentiation trajectories to SPP1^+^ TAMs and exhibit immunostimulatory activity and better prognostic implications

**Figure 5:**
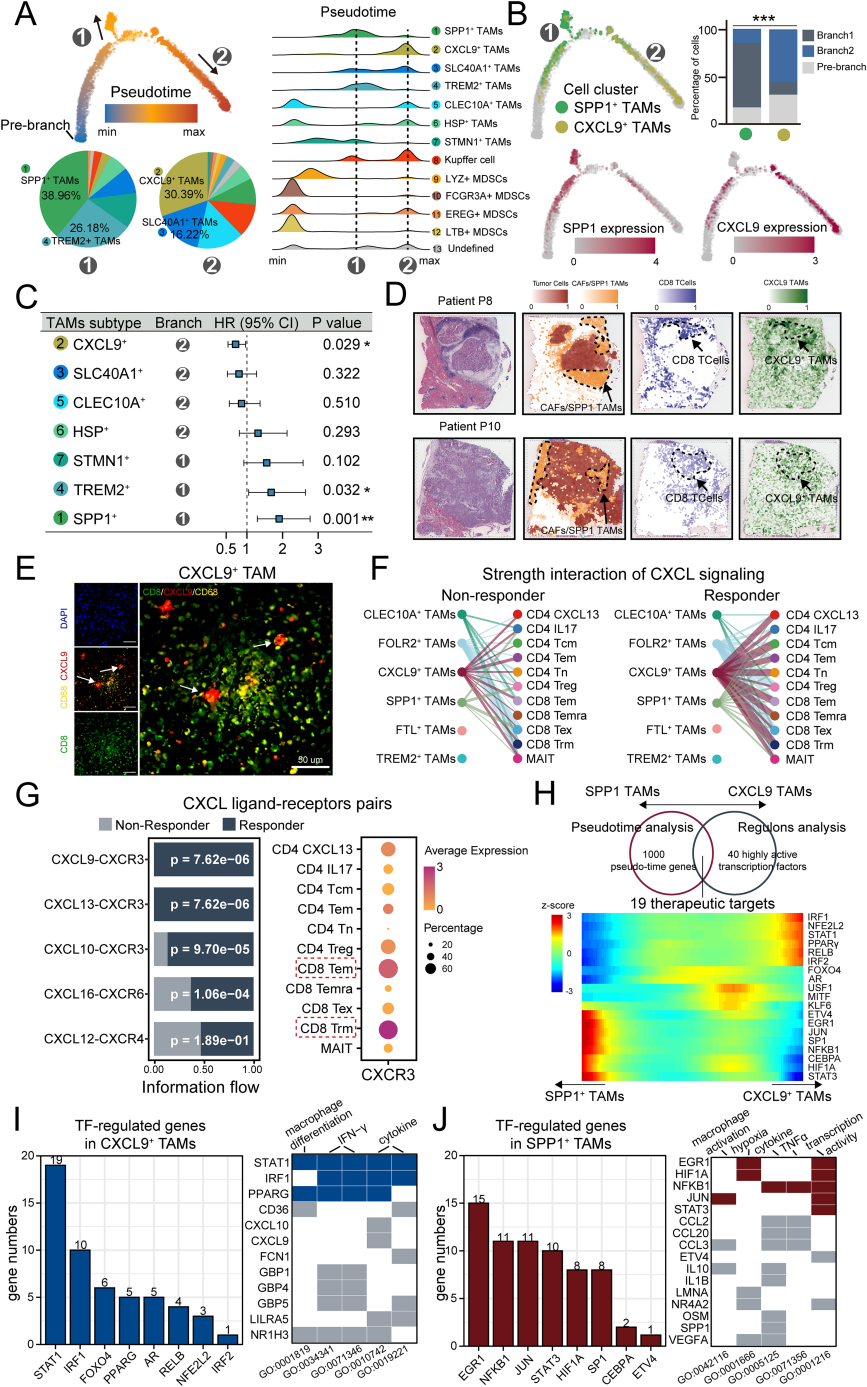
CXCL9^+^ TAM Display Opposing differentiation Trajectories to SPP1^+^ TAMs and Exhibit immunostimulatory Activity and better Prognostic Implications. **(A)** Semisupervised pseudotime trajectory of TAM subtypes by Monocle2. Left: The trajectory is colored by pseudotime (top), and the pie charts indicating the proportion of cells in two developmental branches (bottom). Right: Ridge plot of densities of cell numbers in macrophage subtypes over pseudotime. **(B)** The trajectory is colored by CXCL9 TAMs and SPP1 TAMs clusters (top left), and expression dynamics of two marker genes CXCL9 and SPP1 (bottom). The cell proportion of SPP1 TAMs and CXCL9 TAMs in the two branches was shown by bar plot (top right). **(C)** Forest plot showing the clinical relevance of TAM clusters revealed by cox regression based on overall survival. **(D)** Scores of the CD8^+^ T cells and CXCL9 TAMs signatures enrichment in each spot on ST sections. **(E)** Representative mIF staining of liver tissues from the HCC mouse model. DAPI (blue), CD68 (yellow), CXCL9 (red), CD8 (green) are shown, along with individual and merged channels. Scale bar, 50 μm. **(F)** Hierarchy plot of the CXCL signaling pathway, depicting cell-cell interactions between TAM subtypes (source) and T cell subtypes (target cells) in immunotherapy-responding (left) and immunotherapy-non-responding patients (right). The width of edges represents the strength of communication. **(G)** Left: Relative contribution of each ligand-receptor pair to the overall communication network of CXCL signaling pathway. Right: Dot plot of CXCR3 expression in T cell subtypes. **(H)** Venn diagram showing specific therapeutic targets of CXCL9-SPP1 TAMs conversion based on pseudotime analysis and regulons analysis (top). Heatmap of the therapeutic targets expression changes with pseudotime in CXCL9-SPP1 TAMs conversion (bottom). **(I, J)** Left: Bar chart illustrating the numbers of marker genes positively regulated by TFs in CXCL9^+^ TAMs **(I)** and SPP1^+^ TAMs **(J)**, with a threshold of R > 0.2 and *P* < 0.05. Right: heatmap displaying the representative biological pathways involving TFs and their regulated genes in CXCL9^+^ TAMs **(I)** and SPP1^+^ TAMs **(J)**. Tiles in blue and red indicate TFs, and in gray indicates the genes regulated by these TFs. *<0.05, **<0.01, ***<0.001.

To better understand the dynamics of SPP1^+^ TAMs, we performed pseudotime trajectory analysis to estimate individual cell states to allow causal inference of terminally differentiated cells. The result showed a gradual transition of the MDSCs, acting as precursors to macrophages, leading to the emergence of two distinct branches 1 and 2 (**Figure 5A**). The proportions of cell types differed between the branches. SPP1^+^ TAMs were enriched at the end of branch 1 with the highest proportion of cells (39.86%), while CXCL9^+^ TAMs were enriched in branch 2 (30.39%) (**Figure 5B**). The expression of key markers was coupled with the transition. Interestingly, we observed the divergent prognostic effects in the two branches (**Figure 5C**). The better effect was observed in branch 2, which is consistent with the prognostic value of CXCL9^+^ TAMs (**Figure 3C**). This preliminary observation stimulated our interest in exploring the role of CXCL9^+^ TAMs.

Based on enrichment analysis (**Figure 4A**), CXCL9^+^ TAMs were uniquely and significantly enriched in lymphocyte chemotaxis and T cell proliferation, which was associated with immunostimulatory activity. Based on spatial transcriptomics data, enrichment score with T cell and CXCL9^+^ TAMs signatures highlighted co-localization in the same spot (**Figure 5D**, **Supplementary Figure 4A**). Furthermore, the signature score of T cell and CXCL9^+^ TAMs signatures in spots showed a significantly positive correlation (**Supplementary Figure 4C**). mIF staining demonstrated that CXCL9-positive and CD8-positive T cells were in close proximity (**Figure 5E**, **Supplementary Figure 4E**). To further validate the immunostimulatory role of the CXCL9^+^ TAMs, we analyzed a scRNA-seq dataset of HCC patients receiving immunotherapy with differential response outcomes (**Supplementary Figures 3F–I**). We predict receptor-ligand interactions between subtypes of TAMs and T cells. We focused on the CXCL signaling pathway network because chemotactic signals (CXCL signal) regulate the recruitment and paracrine signal transduction for immune cell development (65, 66). As a result, compared to non-responders, responded tumors displayed more predicted interactions between CXCL9^+^ TAMs and the T cell compartment (**Figure 5F**), and the CXCL9/10/11 and CXCR3 ligand-receptor pairs were significantly enriched in responders (**Figure 5G**), and CXCR3 was prominently expressed in several activated T cell subtypes to function immunostimulatory role (**Figure 5G**).

Finally, we investigated the key regulators affecting the formation of CXCL9^+^ TAMs and SPP1^+^ TAMs. Based on the intersection of the results on pseudotime analysis and regulons analysis, 19 differentially expressed regulators were enriched during the conversion of CXCL9-SPP1 TAMs, which could be potentially therapeutic targets for TAMs therapy (**Figure 5H**). In these factors, like Interferon Regulatory Factor 1 (IRF1) and Nuclear factor, erythroid 2 like 2 (NFE2L2) have been reported to function in the anti-tumor capacity (67, 68), while HIF-1 alpha (HIF1A) and Signal transducer and activator of transcription 3 (STAT3) to support tumor growth and immune evasion (69, 70). We further investigated the expression correlation between these transcription factors and marker genes specifically in CXCL9^+^ TAMs and SPP1^+^ TAMs. Our results showed that in CXCL9^+^ TAMs, STAT1 and IRF1 positively regulated a greater number of genes and were primarily involved in functions such as macrophage differentiation and IFN-γ signaling (**Figure 5I**). Conversely, in SPP1^+^ TAMs, EGR1 and NFKB1 were predominantly involved in regulation and were associated with macrophage activation and transcriptional activity. And these factors also play a role in modulating hypoxic processes (**Figure 5J**). The differential regulation underscored the distinct roles of TAM subsets in the tumor ecosystem and highlighted the complexity of their interactions with the tumor micro-environment. In summary, CXCL9^+^ TAMs display opposing differentiation trajectories to SPP1^+^ TAMs and exhibit immunostimulatory activity and better prognostic implications.

### 3.5 CXCL9:SPP1 (CS) polarity exhibits diverse anti/pro-tumor micro-environment

**Figure 6:**
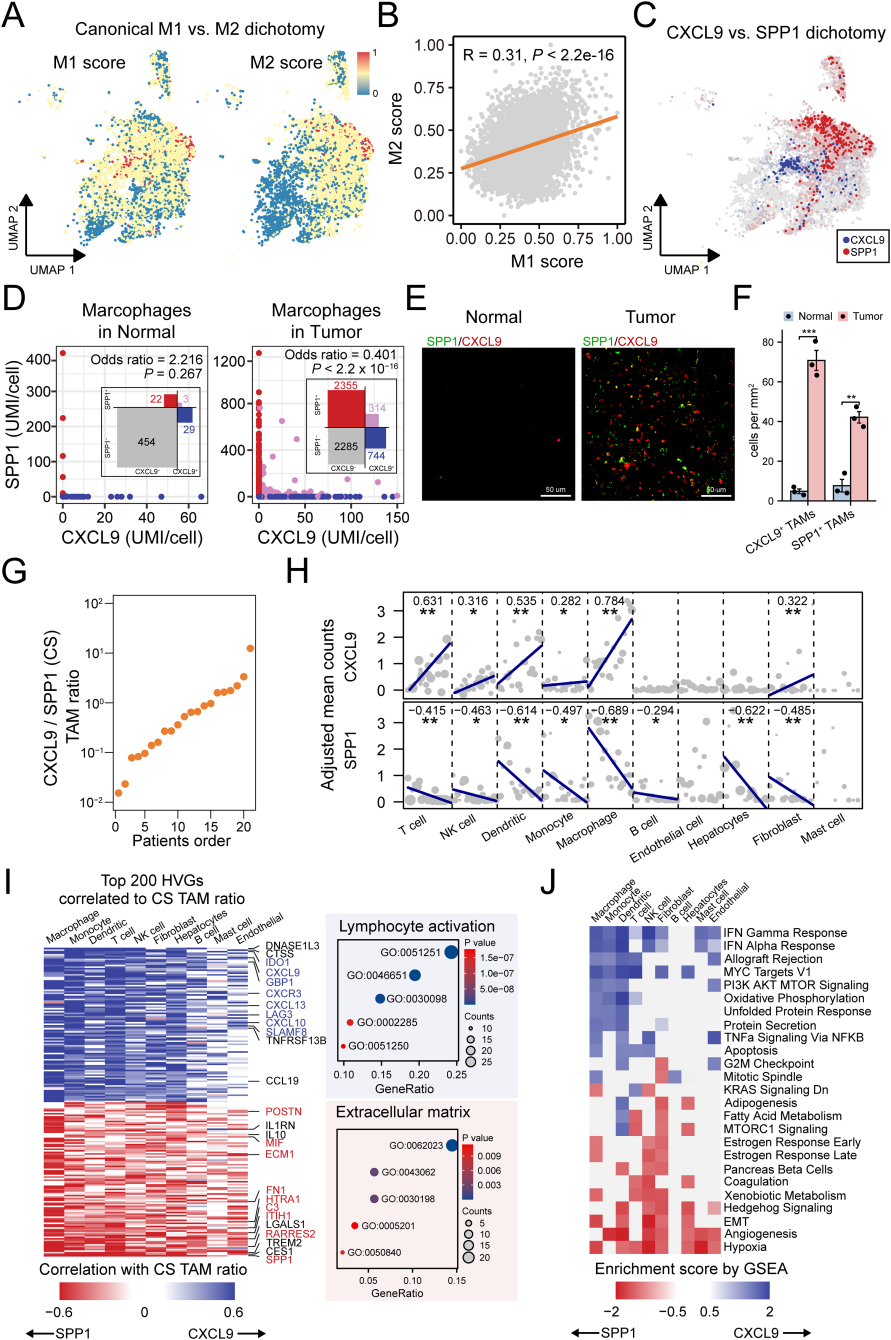
CXCL9:SPP1 TAM polarization exhibits diverse anti/pro-tumor micro-environment. **(A)** Overlay of the expression of common M1 and M2 signature scores in the TAM subtypes. **(B)** Scatter plots showing the correlations between common M1 and M2 signature scores. **(C)** UMAP shows mutually exclusive expression of CXCL9 and SPP1 in TAMs. **(D)** Scatter plot of CXCL9 and SPP1 expressions in TAMs and a contingency table based on dichotomized expression with odds ratio and Fisher’s exact test to indicate mutual exclusion. **(E)** Representative mIF staining for CXCL9^+^ TAMs (red) and SPP1^+^ TAMs (green) in liver sections from mice belonging to normal and HCC tumor groups. Scale bar, 50 μm. **(F)** Comparative quantification of CXCL9^+^ TAMs (***P = 0.0002) and SPP1^+^ TAMs (**P = 0.0013) densities(cells/mm^2^) in the liver tissues of normal and tumor groups (n = 3 per group). **(G)** Patient ranking according to the CS TAM ratio. **(H)** Association between CS TAM polarity and the expression of CXCL9 (left) and SPP1 (right) in major cell types. Each dot represents the value for one sample, with the dot size being representative of the cell number contributing to that value. **(I)** Left: Heatmap summarizing the correlation analysis of top 200 highly variable features (HVGs) in major cell types. Right: Bubble plot showing enrichment result of the genes of interest marked in the heatmap. **(J)** Heatmap of gene set enrichment analysis results across major cell types based on genes ordered by CS ratio. *<0.05, **<0.01, ***<0.001.

Recent research has highlighted that the CXCL9:SPP1 (CS) polarity dictates the anti-tumor or pro-tumor phenotype of TAMs. To broaden this understanding, we explored the applicability of CS polarity in HCC tumors. We tested the expression of M1 and M2 markers, which are widely used for the classification of TAMs. The M1 and M2 signature genes were co-expression in TAMs (**Figure 6A**), and a significant positive correlation between the signature expression was observed (**Figure 6B**), which was consistent with recent reports showing individual TAMs from human tumors generally express both M1 and M2 genes (24, 71). Furthermore, by examining the blend expression of CXCL9 and SPP1 within each TAMs cluster, we found the mutually exclusive expression pattern of CXCL9 and SPP1 in TAMs (**Figure 6C**, **Supplementary Figures 5A, B**), which was confirmed by quantitative analyses in tumors (**Figure 6D**). mIF staining validated that SPP1^+^ macrophages with colocalization of CD68 and SPP1 protein expression, CXCL9^+^ macrophages with colocalization of CD68 and CXCL9 protein expression and were enriched in HCC tumors (**Figures 6E, F**, **Supplementary Figures 5C, D**). Importantly, TAMs subtypes expressed both CXCL9 and SPP1 at different levels (**Supplementary Figure 5E**). Additionally, all subtypes displayed negative correlations between CXCL9 and SPP1 expression, indicating that the CS polarity could be broadly generalizable in HCC (**Supplementary Figure 5F**).

To further understand the role of CS polarity in the TME, we introduced an individual-based method (25) to determine the relationship between CS polarity, the gene expressions and biological function in diverse cell types (**Figure 6G**). The method treats each patient as a distinct statistical unit, calculating the aggregated expression of the CXCL9 and SPP1 genes across various cell types within each unit. By ranking the patients based on the aggregated CXCL9:SPP1 expression ratio of TAMs, we explored the variation in gene expression of other cell types under this ordering scheme. Initially, we found that not only TAMs but also T cells, NK cells, dendritic cells, monocytes and fibroblasts could express CXCL9 (**Figure 6H**). Importantly, all these cell types coordinate with TAMs. For example, patients with higher CXCL9 expressions in TAMs also had higher CXCL9 expression in these other cell types. Similarly, except mast cells, all cell types could express SPP1, and they did so in a coordinated manner with TAMs and in the opposite direction to CXCL9 expression (**Figure 6H**).

Next, we investigated whether the CS polarity affected the transcription levels in other cell types. For this purpose, we analyzed the correlations between the CS polarity and highly variable genes in all TME cell types. We found that 59.1% of genes were significantly associated with CS polarity. We found that CS polarity was mainly positively associated with the expression of cytokines and cytokine receptors for lymphocyte enrollment and activation, while extracellular matrix-related genes expressed more strongly in tumors with lower CS polarity, such as SPP1, RARRES2, C3 and ECM1 (**Figure 6I**). Furthermore, to determine if these genes globally contribute to distinct biological processes, we performed gene set enrichment analyses for each cell type separately, revealing which cell types these programs are active in. Immune-related IFNγ and IFNα signaling, associated with higher CS polarity, was active in nearly all cell types. In contrast, pathways related to lower CS polarity, such as hypoxia and angiogenesis signaling, were present in almost all cell types (**Figure 6J**). Taken together, these findings link CS polarity to multiple immune and non-immune cell processes, influencing tumor growth by either promoting or inhibiting it.

### 3.6 CS polarity served as a potential biomarker for prognosis and therapy outcomes reflection in HCC

**Figure 7:**
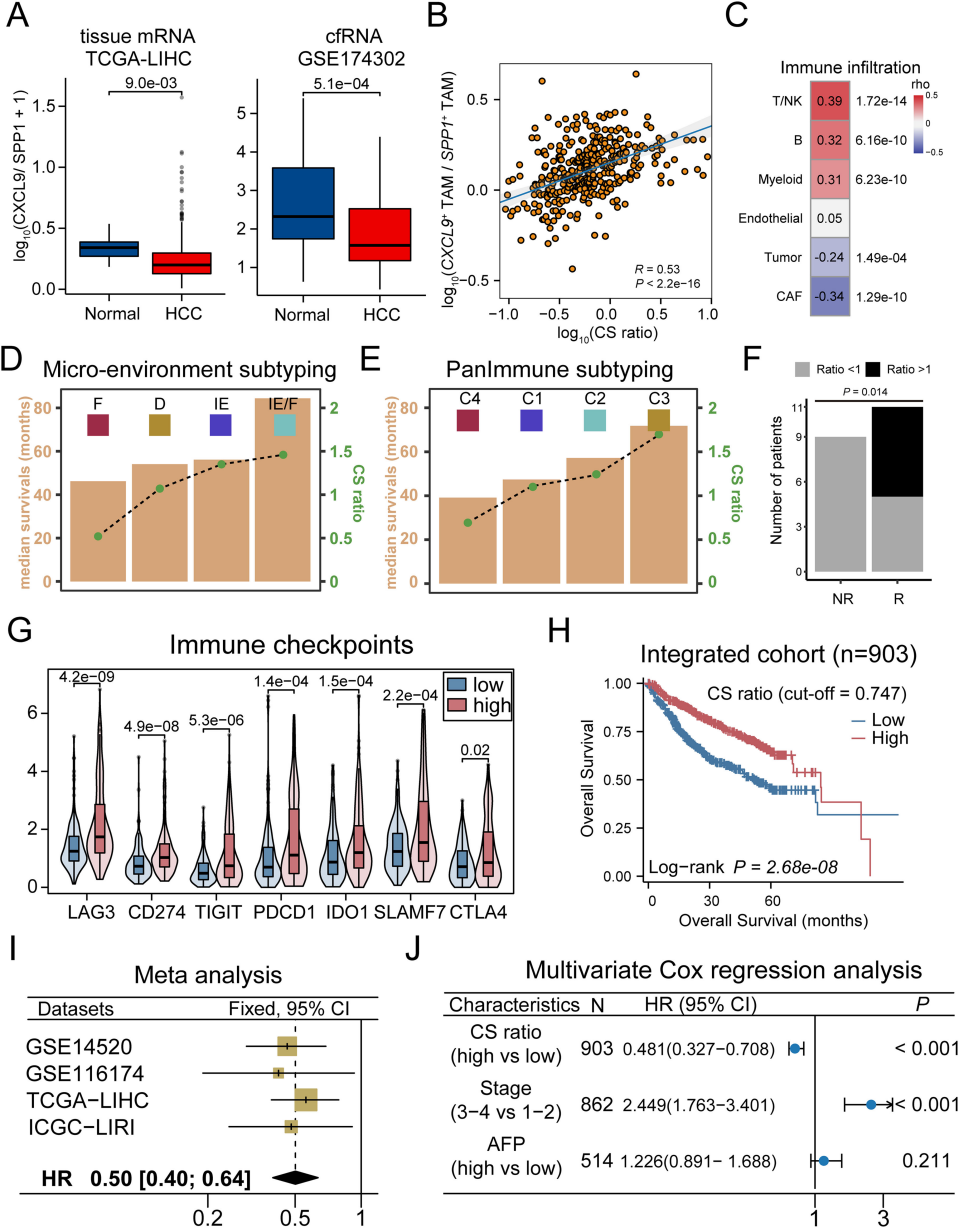
CXCL9/SPP1 polarization level served as valid biomarker for prognosis and therapy outcomes reflection in HCC. **(A)** Left: Violin plots compare CS ratio level in tumor and normal adjacent tissue in TCGA-LIHC cohort. Right: CS ratio level across tumor and normal samples in cfRNA cohort. **(B)** Scatter plot to compute correlation between the CS level and the ratio of CXCL9^+^/SPP1^+^ TAMs scores. **(C)** Correlations between the CS level and major cell types. **(D, E)** The correlation between the overall survival times (yellow bar) and the CS ratio (gray lines with green dots) in micro-environment subtyping **(D)** and PanImmune subtyping **(E)**. **(F)** Association between CS ratio and response to immunotherapy treatment, with significant Fisher’s exact test. **(G)** The expression of immune checkpoint genes in CS^high^ and CS^low^ groups. **(H)** Kaplan-Meier survival curves displaying the OS between groups in combined cohorts. **(I)** Forest plot of meta-analysis on CS ratio levels. **(J)** Patient with higher CS ratio levels display greater survival based on multivariate cox regression analysis.

We further validated the pattern of CS polarity in independent cohorts. Power analysis confirmed that the chosen sample size in each cohorts provide sufficient statistical power of 0.8 and a false discovery rate of 0.05 for detecting differential expression between patients with high and low CXCL9/SPP1 polarity (**Supplementary Figures 2C, D**). Considering the coordinated display of the CS polarity in the micro-environment, we used the CXCL9/SPP1 gene expression ratio to represent this polarity in patients. Differential expressions of CXCL9 and SPP1 were observed in a variety of cancers (**Supplementary Figure 6A**). In HCC, the CS polarity differed significantly between tumor and normal tissue, which is further confirmed at cell-free RNAs (**Figure 7A**). A significant positive correlation was observed between the CS polarity and the ratio of CXCL9^+^ and SPP1^+^ TAMs infiltration level (**Figure 7B**). Consistently, except for endothelial cells, all major cell types showed correlation with the CS polarity (**Figure 7C**), indicating that the CS polarity could reflect the TME infiltration. We divided the patients into CS^high^ and CS^low^ groups based on the median of the CS polarity (**Supplementary Figure 6B**). The 353 differentially expressed genes were identified, and the T-cell related genes (CD8A, CD5L) and chemokines genes (CXCL9, CXCL10, CXCL13) were highly expressed in the CS^high^ group, while mucin gene (MUC13), extracellular matrix remodeling genes (SPP1, MMP7, GPX2) in the CS^low^ group (**Supplementary Figure 6C**). Enrichment analysis reveals significant enrichment in the CS^high^ group involved in T-cell activation, inflammatory, and cytokine production, while fibroblast-related extracellular matrix and WNT signaling were enriched in the CS^low^ group (**Supplementary Figure 6D**).

Molecular subtyping is an effective strategy to identify patients with the poorest prognosis and to design therapeutic strategies for precision medicine (72). We compared the differences in the distribution of consensus subtypes between the groups. As a result, we found that the CS^high^ group had a greater proportion of immuno-enriched (IE) tumors in the TME subtyping and a higher proportion of immunostimulatory (C2) and inflammatory (C3) tumors in the PanImmune subtyping, which is associated with increased T cell activity and longer overall survival. In contrast, CS^low^ group exhibited a higher proportion of fibroblast-enriched (F) tumors in the TME subtyping and a higher proportion of immunodepleted (C4) tumors in the PanImmune subtyping, which showed an elevated expression of angiogenesis and fibroblast pathways with poorer survival outcomes (**Figures 7D, E**, **Supplementary Figures 7A, B**). To support this observation, we calculated the correlation between the TME scores and the CS polarity levels across samples. As a result, the anti-tumor immune infiltration and angiogenic fibroblast pathways were significantly correlated with the scores (**Supplementary Figure 7C**). Consistently, a higher score indicated a more active anti-tumor immune state, while a lower score made patients a more pro-tumor state facilitated by fibroblasts through the formation of the extracellular matrix. In addition, single-cell analysis confirmed that the patients with the lower CS polarity exhibited ineffective treatment outcomes, while those with polarity exceeding 1.0 exhibited efficacious responses to immunotherapy, and cytotoxicity is positively correlated with the CS polarity in patients (**Figure 7F**, **Supplementary Figures 7D, E**). The expression levels of immune checkpoint genes (CD274, CTLA4, LAG3) were significantly higher in patients in CS^high^ group (**Figure 7G**).

Finally, we analyzed the relationship between CS polarity and the prognosis in HCC patients. The CS^high^ group showed a better prognosis both in the individual cohorts and the combined cohort (**Figure 7H**, **Supplementary Figure 7F**), and the meta-analysis demonstrated that the CS polarity were the robust favorable prognostic marker (**Figure 7I**), which was further validated by multivariate Cox regression analysis (**Figure 7J**). Taking together, these results showed that the CXCL9/SPP1 polarization level could serve as a valid biomarker for prognosis and therapy outcomes reflection in HCC.

### 3.7 CS polarity could be measured by CT-based radiomics model

**Figure 8:**
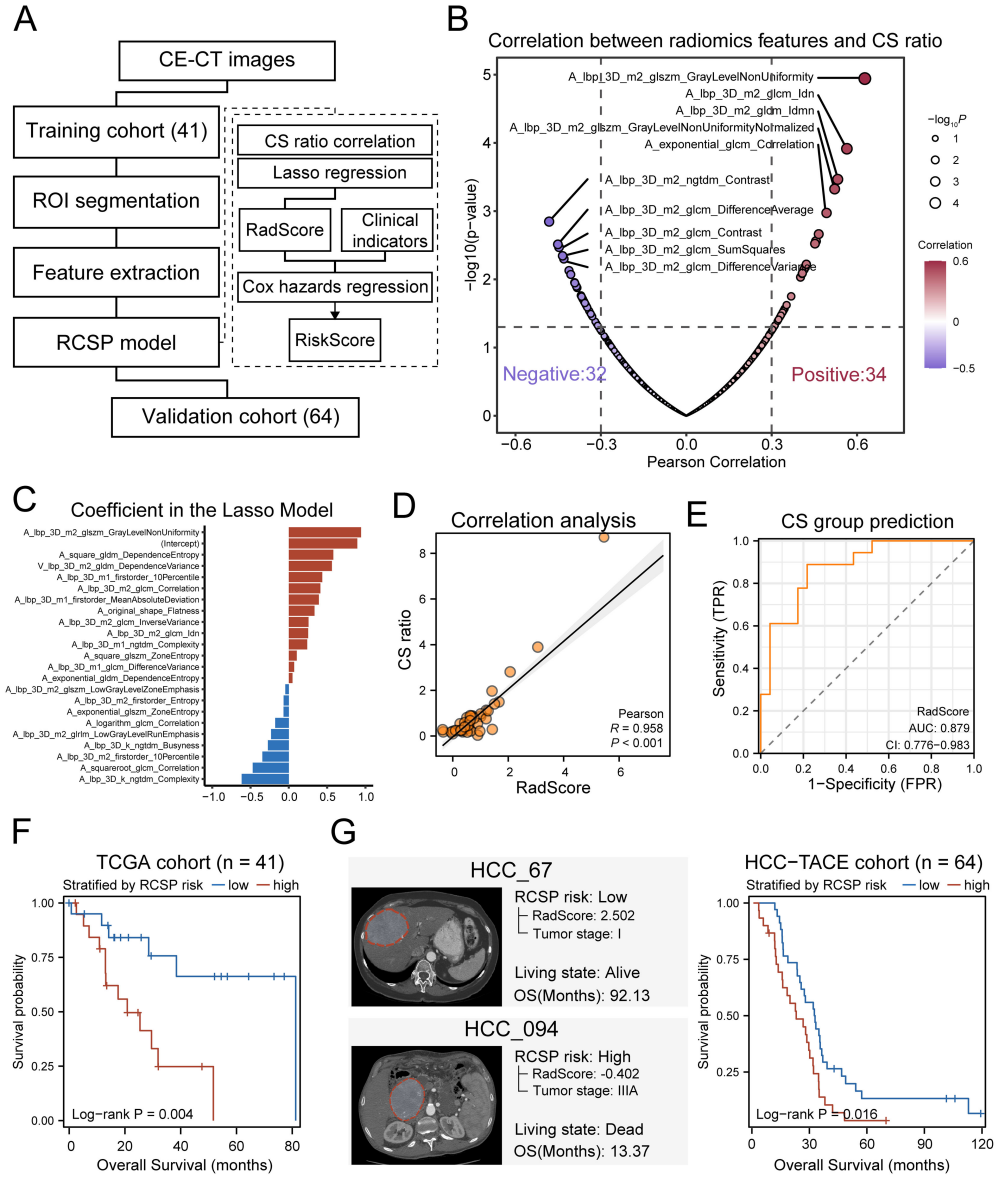
CT-based radiomics model predicts CS ratio levels in HCC. **(A)** Schematic diagram of the radiomics analysis process. **(B)** Volcano plot of the correlation coefficients between radiomics features and the level of CS ratio in HCC patients. Red represents positive correlation, and purple represents negative correlation. The larger dots indicate more significant *P*-values. **(C)** Distribution of coefficients for variables after LASSO regression selection. **(D)** Pearson correlation between the RadScore based on radiomics features and the CS ratio values measured from transcriptome data in the training set. **(E)** ROC curve for predicting the CS ratio status of training set samples based on RadScore. The CS ratio status of samples is grouped by the median of CS ratio in TCGA-LIHC samples. **(F)** Kaplan-Meier curves of overall survival for training set samples grouped by the median of RCSP RiskScore. **(G)** Left: Example of predicted RCSP RiskScore for external validation set samples. Right: Kaplan-Meier curves of overall survival for validation set samples grouped by the median of RiskScore.

Given that the CS polarity effectively reflected the micro-environmental status and the prognostic outcome, it was valuable to explore non-invasive strategies for predicting CS polarity. Two radiomics cohorts (TCIA-LIHC, n=41, TCIA-HCC-TACE-Seg, n=64) were used to construct the prediction model. CE-CT images from the arterial and venous phases were selected as training data. After image quality control, the TCIA-LIHC (n=41) dataset was used as the training set, and the HCC-TACE (n=64) dataset was used as the external validation set. The radiomics analysis process is outlined in **Figure 8A**, which includes tumor segmentation, feature extraction, RCSP (Radiomics-based CXCL9/SPP1 Polarity) model building, and prediction on the validation set.

After feature extraction and normalization, out of 400 radiomics features, 66 features were found to have significant correlations with the CS polarity of patients in the training set measured by the transcriptome based on Pearson’s correlation (r > 0.3, *P* < 0.05) (**Figure 8B**). Using LASSO regression for dimension reduction, a final set of 22 radiomics features was selected to establish a predictor (RadScore) to the CS polarity in patients (**Figure 8C**, **Supplementary Figures 8A, B**). Most of the features were texture features, primarily from the arterial phase (**Supplementary Figure 8C**). The CS polarity predicted by the RadScore showed significantly positive correlations with its levels measured by the transcriptome in the training set (r=0.958, p=1.3×10^–9^, **Figure 8D**). The ROC value for predicting patient CS polarization groups based on radiomics reached 0.879 (**Figure 8E**), indicating that the RadScore accurately reflected the CS polarity of patients well.

To further enhance prognostic robustness and clinical value, we combined RadScore with clinical indicators to construct the RCSP model. After univariate Cox regression analysis, tumor grade was the clinical variable that met the inclusion criteria, along with RadScore (**Supplementary Table 3**). These variables were incorporated into a Cox proportional hazards regression model to calculate a risk score (RiskScore) for each patient. Survival analyses showed that RiskScore tended to stratify the overall survival of HCC patients (log-rank *P* = 0.004) (**Figure 8F**). The validation cohort confirmed the applicability of the RCSP model. Patients classified as high-risk showed markedly poorer survival outcomes (log-rank *P* = 0.016) (**Figure 8G**), confirming the prognostic value of the RCSP model. These findings demonstrate not only that the macroscopic effects of CS polarization can be observed using CT but also that the RCSP model provides a non-invasive method for predicting CS levels and patient prognosis.

Finally, a radiomics tool was developed to implement RCSP model (https://github.com/YuGu-CN/RCSP) based on the R programming language. It encompassed essential functions including image format conversion, tumor section preview, and radiomics feature extraction. Additionally, the package integrated the RCSP model that estimated the CS polarity in HCC patients based on extracted radiomics features. Users could prepare CE-CT images, and the package would automatically handle format conversion and feature extraction, subsequently generating a concise report file (**Supplementary Figure 8D**).

## 4 Discussion

Altering TAMs phenotype is a new potential therapeutic approach to activate anti-tumor immunity. TAMs are heterogeneous cell populations with different phenotypes that promote immune evasion, tumor growth, and metastasis. Simple M1/M2 dichotomization of macrophage subtypes cannot perfectly profile the diversity of TAMs phenotypes, highlighting the necessity for a more rational classification of TAMs populations (73). Some studies have identified TAMs subtypes with distinct tendencies toward tumor suppression or promotion. In this study, we focused on multi-regional scRNA-seq data and employed an integrated analysis approach to identify key cell types that impact the progression of HCC. We characterized seven TAMs populations in HCC tissues. Based on three computational methods, we identified SPP1^+^ TAMs as a critical subset in the progression of HCC. Compared to other subsets, SPP1^+^ TAMs are extensively increased in tumor tissues and are more likely to be enriched in the tumor core, suggesting that they may play a key role in promoting tumor growth. SPP1, a secreted non-collagenous glycoprotein, is an important adhesion protein and cytokine (74). Recently, SPP1 has been shown to be expressed and secreted by TAMs in various cancers, promoting macrophage polarization, migration, sustained activation, and affecting the cytokine profile of macrophages (75, 76). Based on TCGA-LIHC cohort, we validated that the SPP1^+^ TAMs subset is associated with tumor progression and poor prognosis in HCC.

Accumulating evidence indicates that fibroblasts could interact with various immune components by secreting a variety of chemokines, cytokines, and other biomolecules. This leads to the formation of an immunosuppressive niche that promotes tumor immune evasion (77, 78). Through functional analysis, we found that SPP1^+^ TAMs did not perform common antigen-presenting functions but are involved in the extracellular matrix (ECM) remodeling, which makes us suspect their functional promotion with cancer-associated fibroblasts (CAFs). We identified CAFs associated with poor outcomes in HCC patients and showed the strongest infiltration correlation with SPP1^+^ TAMs. Furthermore, through cell communication and spatial transcriptomics analysis, we demonstrated that these cells form spatial co-localization based on ligand-receptor interactions and promote the formation of ECM structures. Consistently, previous studies have found that the interaction between SPP1^+^ TAMs and fibroblasts contributes to the formation of a tumor immune barrier, promotes the accumulation of ECM structures, and is important for the efficacy of immunotherapy (30). These findings also prove that the interaction between SPP1^+^ macrophages and CAFs is an important factor affecting the phenotype of malignant tumors and leading to worse outcomes.

Through pseudotime trajectory analysis, we identified the cellular states of TAMs subpopulations and delineated the evolutionary dynamics of these subpopulations. We discovered that the TAMs subpopulations formed two distinct branches, with CXCL9^+^ TAMs displaying opposing differentiation trajectories to SPP1^+^ TAMs. The CXCL9^+^ TAMs were associated with survival advantages, suggesting that CXCL9 might be an integral component of antitumor activity. The role of CXCL signaling in T cell recruitment has been described in other cancer types (54, 79). Using spatial transcriptomics data, we found significant spatial colocalization between CXCL9^+^ TAMs and T cells. Cell communication analysis based on single-cell data from immunotherapy revealed that the CXCL signaling was highly expressed in CXCL9^+^ TAMs of patients responding to immunotherapy, and the main targets of CXCL9/10/11, CXCR3, were expressed in T cells, which are the proposed effectors of direct antitumor cytotoxicity within the TME (80). A growing body of evidence points to the CXCR3 chemokine pathway as a significant axis of anti-PD(L)-1 therapy response, regulating the recruitment and positioning of effector T cells within the TME (80, 81). Interferon (IFN)-induced CXCR3 ligands, CXCL9/10/11, regulate tumor angiogenesis, enhance T cell infiltration, and further position activated T cells near antigen-presenting cells within the TME, which may provide additional cues to T cells that facilitate antitumor immunity (82).

The early dichotomy paradigms of TAMs polarization, characterized by pro-inflammatory M1 and anti-inflammatory M2, were identified by similar surface marker expressions. Using scRNA and ST technique, novel identified subsets of TAMs have exhibited phenotypes not belonging to M1 and M2 classifications or simultaneously expressed those markers (23, 24). Therefore, a more complete mechanistic understanding of TAMs polarization is needed in cancer. Seminal findings by Pittet et al. highlight the superior utility of CXCL9:SPP1 polarity compared to the M1 and M2 markers (25, 26). To better understand the phenomenon, we explored for the first time the applicability of CXCL9:SPP1 polarity in HCC tumors. The expression of CXCL9 and SPP1 was observed to be mutually exclusive across all TAMs subsets in HCC, with co-expression being a rare occurrence, suggesting that each subset more-or-less retained the potential of shaping opposite phenotypes of CXCL9^+^ and SPP1^+^ TAMs. We introduced a population-centric approach to analyze how CS polarity influences the overall gene expression landscape and biological functions within TME. Nearly 59.1% of highly variable genes within TME exhibited significant correlations with CS polarity. Further functional analyses revealed highly correlation between CS polarity and immune-related (IFNγ,IFNα and cytokines) along with oncogenic signaling (hypoxia and angiogenesis), suggesting an anti-tumor or pro-tumors status globally. Importantly, we sought to detect several key transcription factors involved in shaping CS polarity in HCC. It is possible that STAT1, IRF1, PPARG, EGR1, NFKB1 and JUN may play an essential role in this process, as they regulate more markers related to CS polarity compared to other regulators. These transcription factors are implicated in macrophage differentiation or activation, playing a dual role in regulating either a pro-tumor or anti-tumor response. Interestingly, these factors are not entirely within the known range of transcription factors that intervene in M1 or M2 polarization, which could provide new therapeutic targets or new insights for the reprogramming of targeted macrophages (83). Of course, this conjecture requires further experimental confirmation.

Our findings suggest that CS polarity plays a critical role in shaping the tumor microenvironment and influencing HCC prognosis. Patients with high CS scores have better prognosis and respond better to immunotherapy. However, we observed that patients with low CS scores exhibited a higher abundance of SPP1^+^ TAMs, which are associated with an immunosuppressive microenvironment and tumor progression. According to the microenvironment subtyping proposed, tumors classified as fibrotic subtype are characterized by a dense fibrotic stroma and are recommended for anti-angiogenic and anti-fibrotic therapies. In our study, we found that patients with low CS scores were more frequently classified into this fibrotic subtype (**Supplementary Figure 6A**), further supporting the potential applicability of these treatment strategies in this patient subgroup. Beyond existing therapeutic approaches, our findings highlight the need for targeting SPP1^+^ TAMs as a potential intervention for patients with low CS scores. Future studies could explore strategies aimed at modulating the epigenetic regulation of these TAMs, such as inhibiting key transcription factors or disrupting regulatory networks that sustain their polarization. Additionally, reprogramming SPP1^+^TAMs into CXCL9^+^ TAMs may offer an alternative approach to enhance anti-tumor immunity and reshape the immune landscape in HCC. Further investigation into these regulatory mechanisms may provide novel therapeutic avenues for patients with unfavorable CS polarity.

In recent years, radiomic technology has become an important direction in HCC research. Radiomics uses high-throughput methods to analyze medical imaging data, which can reveal the micro and macro characteristics of tumors, thereby helping to better understand and treat HCC (84, 85). Previous studies have developed many CT-based or MRI-based radiomics biomarkers for immune infiltration, although the reported radiomics features are heterogeneous and have limited reproducibility (86–88). We validated the widespread presence of CXCL9/SPP1 in bulk data across multiple cohorts, proving that the CXCL9/SPP1 ratio may serve as a biomarker reflecting the state of the micro-environment and different prognostic outcomes in HCC patients. Here, we attempt to establish the first radiomics biomarker for the tumor-infiltrating CS polarization state, thereby demonstrating its extensive impact on the TME. Under the condition of limited patient numbers in the imaging genomics data, we used a conservative algorithm based on linear correlation and L1 norm for feature selection to reduce the risk of overfitting. The results show that the selected CT radiomic features can serve as indicators of the CS polarity in patients, thereby implying that the CS polarity has a significant impact on the tumor and its micro-environment. In addition, we have developed a package that can predict the CS polarization state and further reflect the survival status of patients using CT imaging data. This helps to identify high-risk individuals and achieve precise diagnosis and personalized treatment.

Our findings highlight the potential of CS polarity as a prognostic and therapeutic biomarker in HCC. However, its clinical application requires further exploration. Several aspects warrant discussion. (1) Potential as a Biomarker for Immunotherapy Stratification. Given the distinct functional roles of CXCL9^+^ and SPP1^+^ TAMs in shaping the tumor microenvironment, CS polarity could serve as a potential biomarker for stratifying patients in immunotherapy. CXCL9^+^ TAMs are associated with an immunostimulatory microenvironment, while SPP1^+^TAMs contribute to an immunosuppressive niche. Future studies could assess whether patients with a predominant CXCL9^+^ TAM profile may benefit more from immune checkpoint inhibitors or other immunotherapies. (2) Development of Targeted Therapeutic Strategies. The differential functions of SPP1^+^ and CXCL9^+^ TAMs suggest that targeting these macrophage subsets could be a viable therapeutic approach. Potential strategies may include targeting key transcription factors involved in CS polarity regulation or disrupting super-enhancer-mediated transcriptional programs governing TAM polarization. Further investigations into these mechanisms could facilitate the development of therapies aimed at reprogramming macrophage polarization toward an anti-tumor phenotype. (3) Future Directions in Epigenetic Regulation of CS Polarity. The regulatory landscape of CS polarity remains incompletely understood. Future research could focus on epigenetic modifications, such as histone modifications and DNA methylation patterns, that dictate SPP1 or CXCL9 expression in TAMs. Single-cell epigenomic profiling and CRISPR-based functional screens may help uncover key regulatory elements controlling CS polarity and identify novel therapeutic targets. (4) Challenges and the Need for Large-Scale Clinical Validation. While our study provides novel insights into CS polarity, its clinical translation remains in its early stages. Recent research has begun to explore the relevance of macrophage polarity in cancer prognosis (27), but further validation in large-scale, multi-center cohorts is required. The integration of radiogenomics tools like RCSP into clinical workflows will also necessitate extensive validation to ensure robustness, reproducibility, and clinical feasibility.

## 5 Conclusions

**Figure 9:**
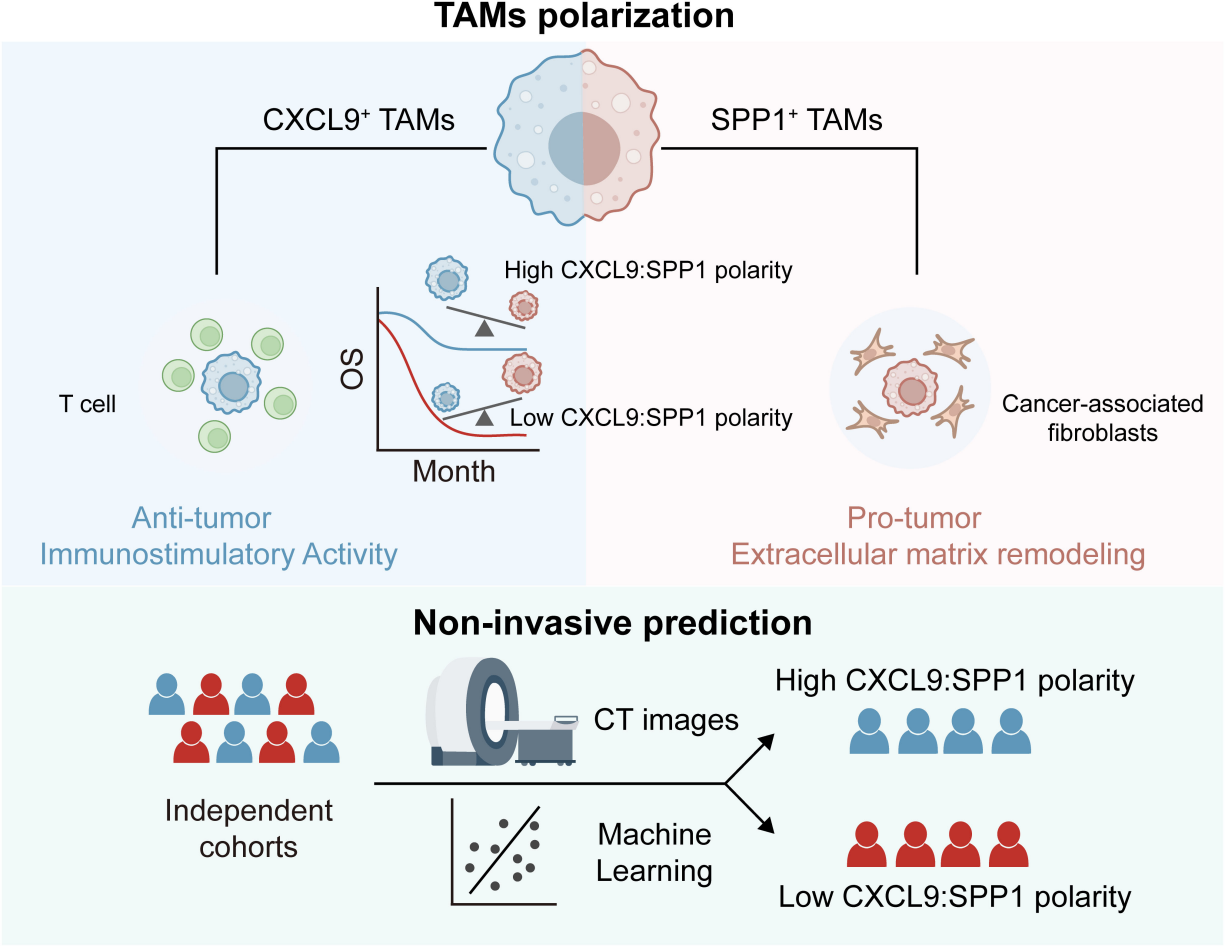
Graphical abstract for the dual role of CXCL9/SPP1 polarized tumor-associated macrophages in modulating anti-tumor immunity in hepatocellular carcinoma.

In summary, our research sheds light on the regulatory roles of SPP1^+^ TAMs and CXCL9^+^ TAMs in the micro-environment and provides new therapeutic targets or insights for the reprogramming of TAMs in HCC. The identification of the CS ratio as a biomarker and the development of a radiomics model for non-invasive diagnosis highlight the clinical potential of targeting TAMs in HCC treatment strategies (**Figure 9**).

## Data Availability

The original contributions presented in the study are included in the article/**Supplementary Material**. Further inquiries can be directed to the corresponding author.
