# Supplementary material for: The dual role of CXCL9/SPP1 polarized tumor-associated macrophages in modulating anti-tumor immunity in hepatocellular carcinoma
Source: Front Immunol. 2025 Mar 31;16:1528103. doi: 10.3389/fimmu.2025.1528103 (PMC11994707; doi:10.3389/fimmu.2025.1528103)
Supplement: Supplementary file 1 [file Image1.pdf]

Supplementary Material

The Dual Role of CXCL9/SPP1 Polarized Tumor-Associated Macrophages in Modulating Anti-Tumor Immunity in Hepatocellular Carcinoma

Yu Gu[1], Zhihui Zhang[2], Hao Huang[1], Wenyong Zhu[1], Hongjia Liu[1], Rongxin Zhang[1], Nan Weng[1], and Xiao Sun[1*]

[1]State Key Laboratory of Digital Medical Engineering, School of Biological Science and Medical Engineering, Southeast University, Nanjing, China.

[2]College of Acupuncture-Moxibustion and Tuina, Nanjing University of Chinese Medicine, Nanjing, China

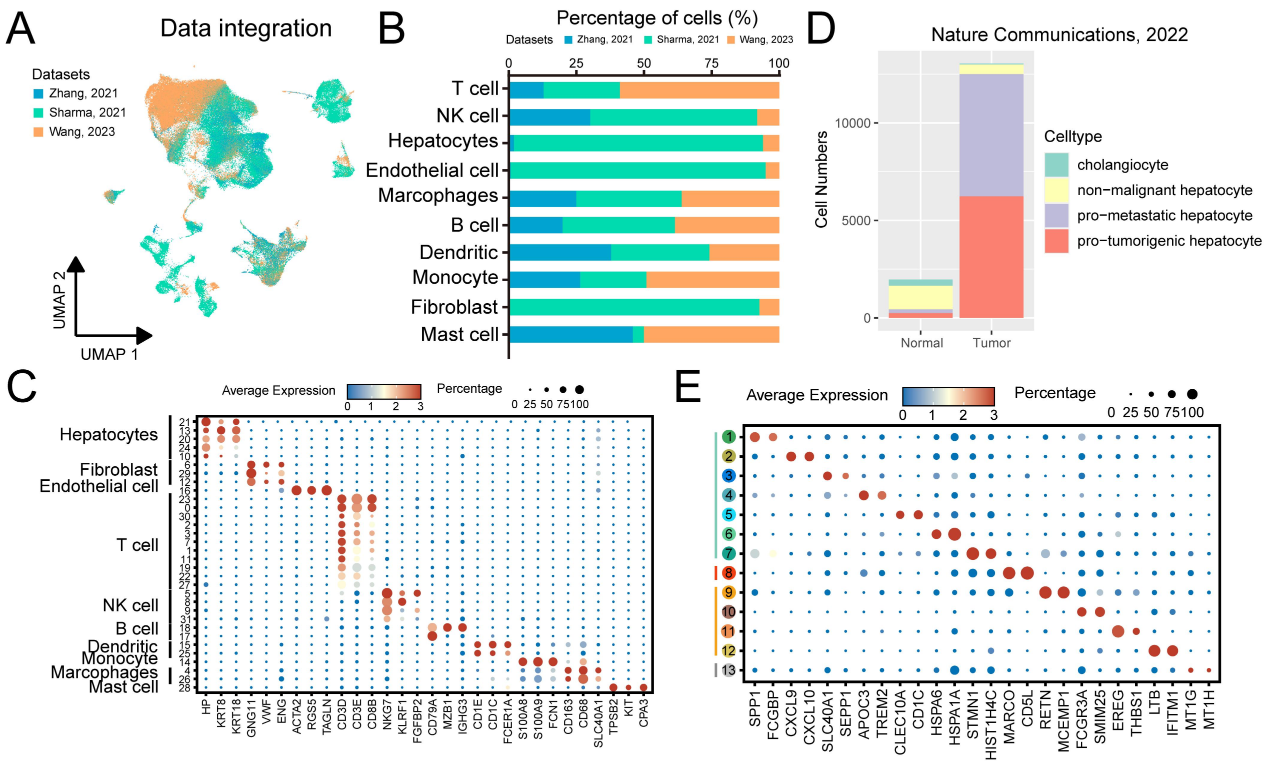

Supplementary Figure 1. Construction of the TAMs transcriptional atlas across multi-region samples. (A) Three datasets merged in this study, batch effect corrected with Seurat integration, and projected onto the bidimensional UMAP space. (B) The proportion of major cell types showing in bar plots in different datasets. (C) Heat map showing relative mRNA expression levels of genes across major celltypes. (D) The stacked bar chart shows the cell count distribution of different

hepatocyte types from an additional study on HCC.(E) Heat map showing relative mRNA levels of marker genes across TAM subtypes.

A

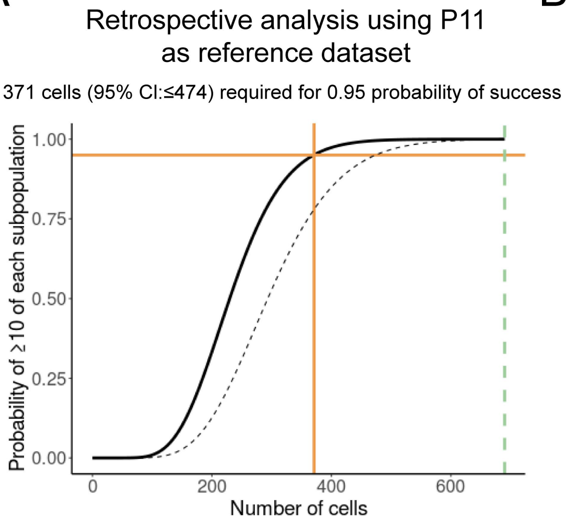

B

Dataset	Sample	Seurat QC filtration	Number of cells in the smallest cell type	Percentage	Retrospective power analysis using control
GSE189903	1H	20575	78(Mast cell)	0.004	Probability of detecting >=10 cells of each subpopulation whenat least 371 (95%CI:<=474) cells are sequenced = 0.95
GSE189903	2H	14472	38(Mast cell)	0.003	
GSE189903	3H	17576	70(Hepatocytes)	0.004	
GSE189903	4H	17160	77(Mast cell)	0.004	
GSE156337	D20171109	6699	32(Endothelial cell)	0.005	
GSE156337	D20180108	8458	33(Fibroblast)	0.004	
GSE156337	D20180110	8018	29(Endothelial cell)	0.004	
GSE156337	D20180116	2776	35(Endothelial cell)	0.013	
GSE156337	P1	2427	30(Hepatocytes)	0.012	
GSE156337	P2	3994	34(Hepatocytes)	0.009	
GSE156337	P3	11238	35(Fibroblast)	0.003	
GSE156337	P4	1700	30(Mast cell)	0.018	
GSE156337	P5	6757	55(Fibroblast)	0.008	
GSE156337	P6	9158	37(Fibroblast)	0.004	
GSE156337	P7	4684	29(Mast cell)	0.006	
GSE156337	P8	5988	86(Fibroblast)	0.014	
GSE156337	P9	5589	29(Mast cell)	0.005	
GSE156337	P10	4760	35(Mast cell)	0.007	
GSE156337	P11	690	42(Fibroblast)	0.061	
GSE156337	P12	5431	30(Mast cell)	0.006	
GSE156337	P13	2033	48(Fibroblast)	0.024	
GSE156337	P14	6986	100(B cell)	0.014	

C

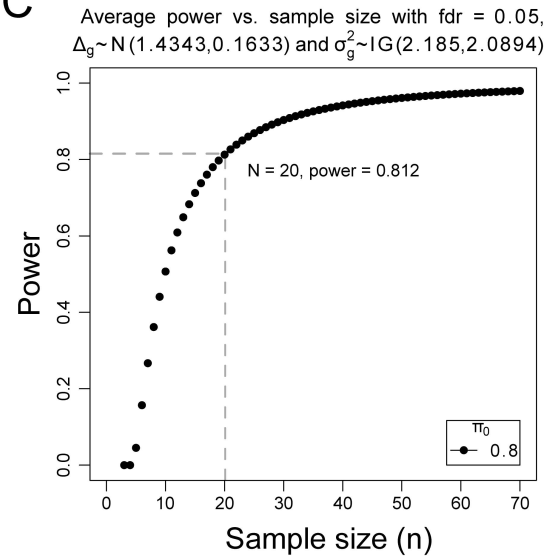

D

Dataset	Data type	Sample	Power analysis
TCGA-LIHC	bulk transcriptomics	377	Minimum of 20 samples in one group was needed
ICGC-LIRI-JP	bulk transcriptomics	232	
GSE14520	bulk transcriptomics	221	
GSE116174	bulk transcriptomics	64	
TCIA-TCGA-LIHC	radiogenomics	41	
TCIA-HCC-TACE-Seg	radiomics	64	

Supplementary Figure 2. Power analysis for multi-omics data. (A) Graph shows the probability of capturing greater than or equal to 10 cells of a rare population with probability of 0.95 with dotted lines showing 95% confidence interval. (B) Table listing the results of retrospective power analysis, and the number of filtered cell after quality control. (C) Power calculation for minimum sample size. To achieve a power of 0.8 with a false-discovery rate of 0.05 to detect 15% of the transcriptome to be differentially expressed, and a minimum of 20 samples in one group was needed. (D) Table listing the sample size in each dataset. Minimum sample size required for each dataset to achieve the desired statistical power (0.8) based on power analysis.

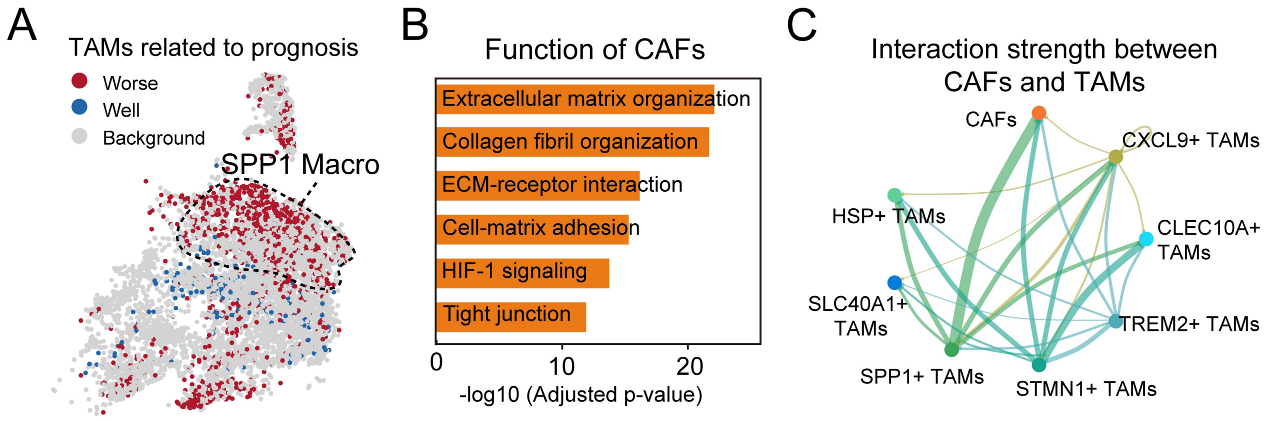

Supplementary Figure 3. SPP1^{+} TAMs and CAFs synergistically contribute to pro-tumorigenic microenvironment in HCC. (A) The UMAP visualization of Scissor analysis of TAMs subtypes. The cells related to poor prognosis are colored in red, and well prognosis in blue. (B) Bar plot showing the enriched GO terms of deferentially expressed genes in CAFs. (C) Chord diagrams showing predicted interaction of TAM subsets with CAFs.

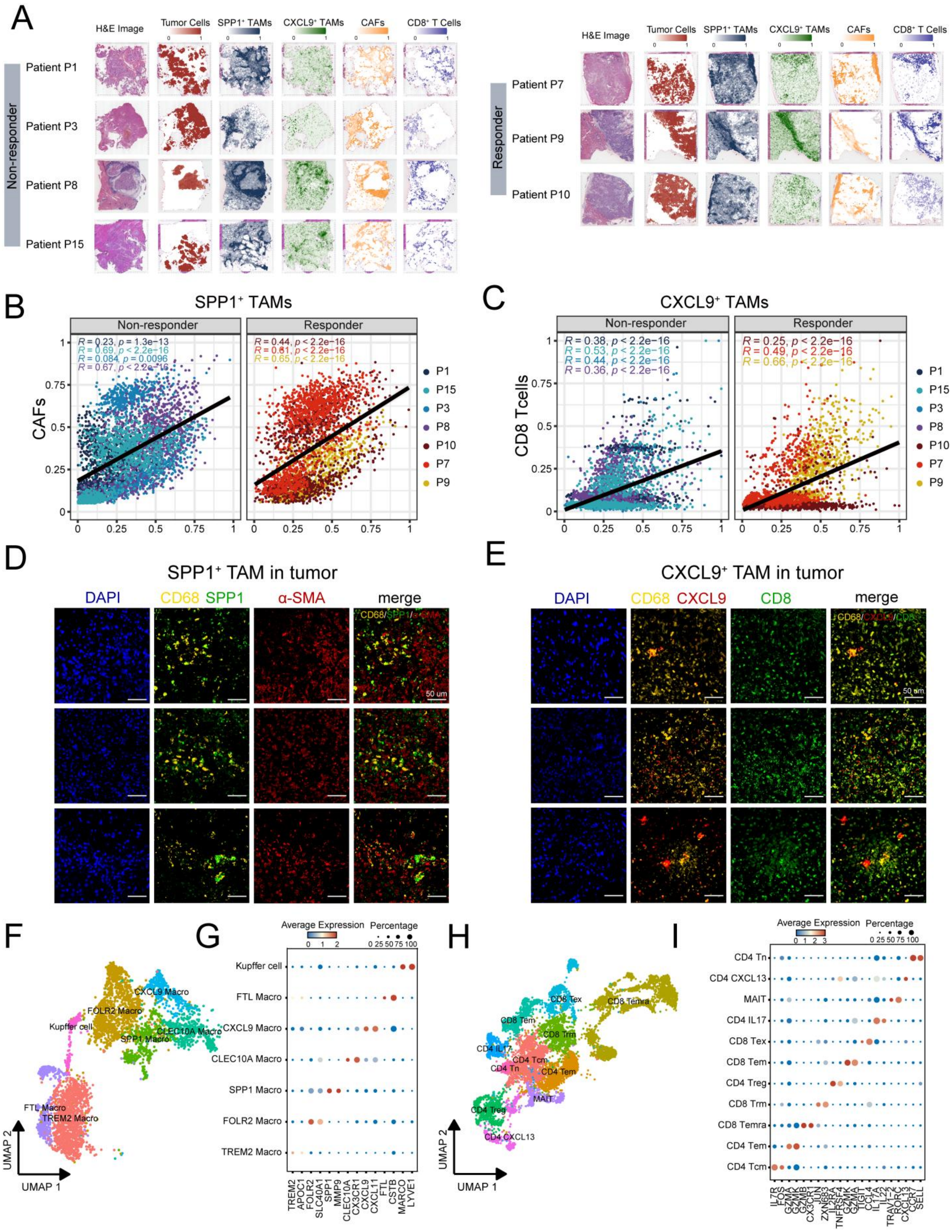

Supplementary Figure 4. The spatial distribution between SPP1$^+$ and CXCL9$^+$ TAMs exhibits marked differences. (A) The first column displays H&E-stained images showing the tumor tissues

from HCC patients receiving immunotherapy on Mendeley Data. In the two to sixth columns, spots are colored by the annotated cell types in scRNA-seq data, tumor or non-tumor regions using the Cottrazm and AddModuleScore method. (B, C) Scatter plots showing the correlation between CAF signature scores and SPP1 TAMs (B), $CD8^+$ T cells and CXCL9 TAMs (C) signature scores in each spatial spot, facet by the outcome of immunotherapy response. (D) Representative mIF staining of liver tissues from the HCC mouse model. DAPI (blue), CD68 (yellow), SPP1 (green), α-SMA (red) are shown, along with individual and merged channels. Sacle bar, 50 µm. n = 3. (E) Representative mIF staining of liver tissues from the HCC mouse model. DAPI (blue), CD68 (yellow), CXCL9 (red), CD8 (green) are shown, along with individual and merged channels. Sacle bar, 50 µm. n = 3. (F, H) UMAP representation depicting macrophages and T cell subpopulations. (G, I) Dot plot showing the marker gene expression levels of macrophages (F) and T cell (H) subpopulations.

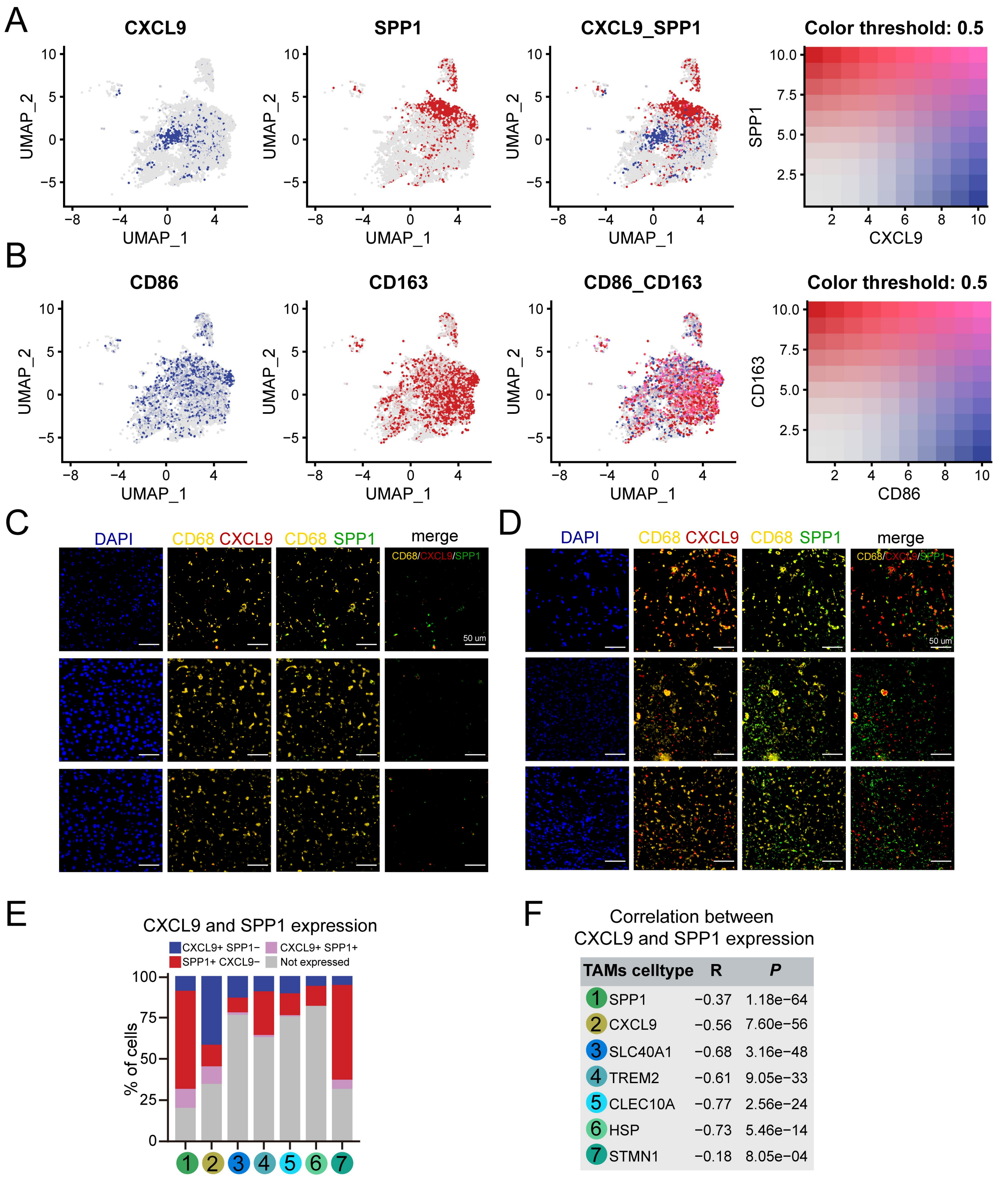

TAMs celltype	R	*P*
1 SPP1	−0.37	1.18e−64
2 CXCL9	−0.56	7.60e−56
3 SLC40A1	−0.68	3.16e−48
4 TREM2	−0.61	9.05e−33
5 CLEC10A	−0.77	2.56e−24
6 HSP	−0.73	5.46e−14
7 STMN1	−0.18	8.05e−04

Supplementary Figure 5. Mutually exclusive expression of CXCL9 and SPP1 in TAMs (A-B) UMAP showing the blend expression of CXCL9 and SPP1 (A) or M1 gene CD86 and M2 gene CD163 (B) in TAMs. (C-D) Representative mIF staining for CXCL9^{+} TAMs and SPP1^{+} TAMs in normal liver tissues (C) and HCC tumur tissues (D) in mouse. DAPI (blue), CD68 (yellow), CXCL9

(red), SPP1 (green) are shown, along with individual and merged channels. Bar, 50 µm. N = 3 per group. (E) Bar plot showing the proportion of cells in dichotomized expression of CXCL9 and SPP1 in per TAM subtypes. (F) The negative correlations between individual expression of CXCL9 and SPP1 was observed in all TAM subtypes.

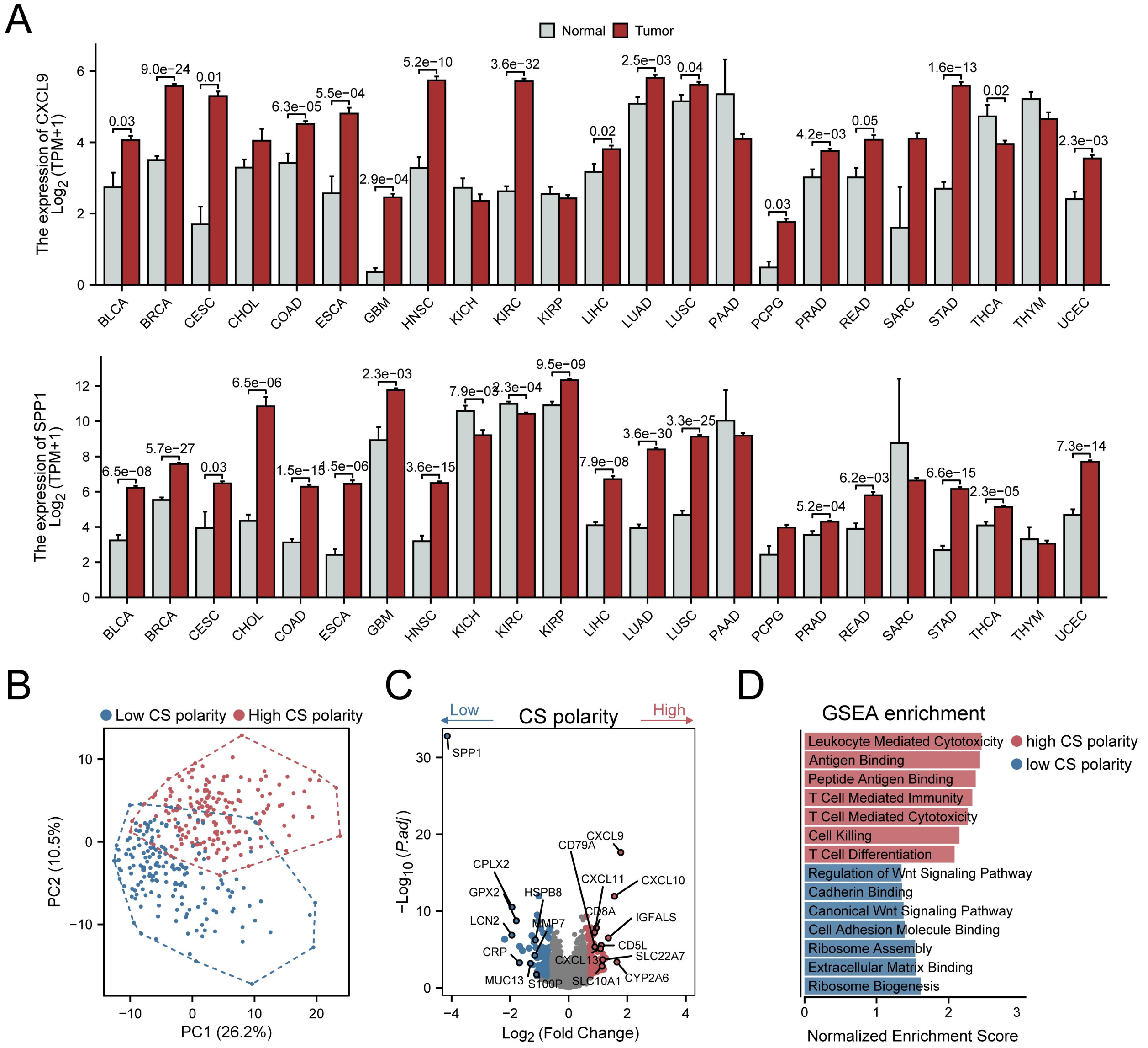

Supplementary Figure 6. The CS polarity are differentially expressed in tumors. (A) The CXCL9 and SPP1 gene expression in pan-cancer tissues and paired non-tumor samples. (B) Principal component analysis plot showing patients with a high or low CS polarity in red or blue, respectively. The patients were grouped based on the median of the CS polarity. (C) Volcano plot showing differential expression genes in high vs. low groups of CS polarity. (D) Gene set enrichment analysis was performed to compare the enrichment of differential expression genes between groups.

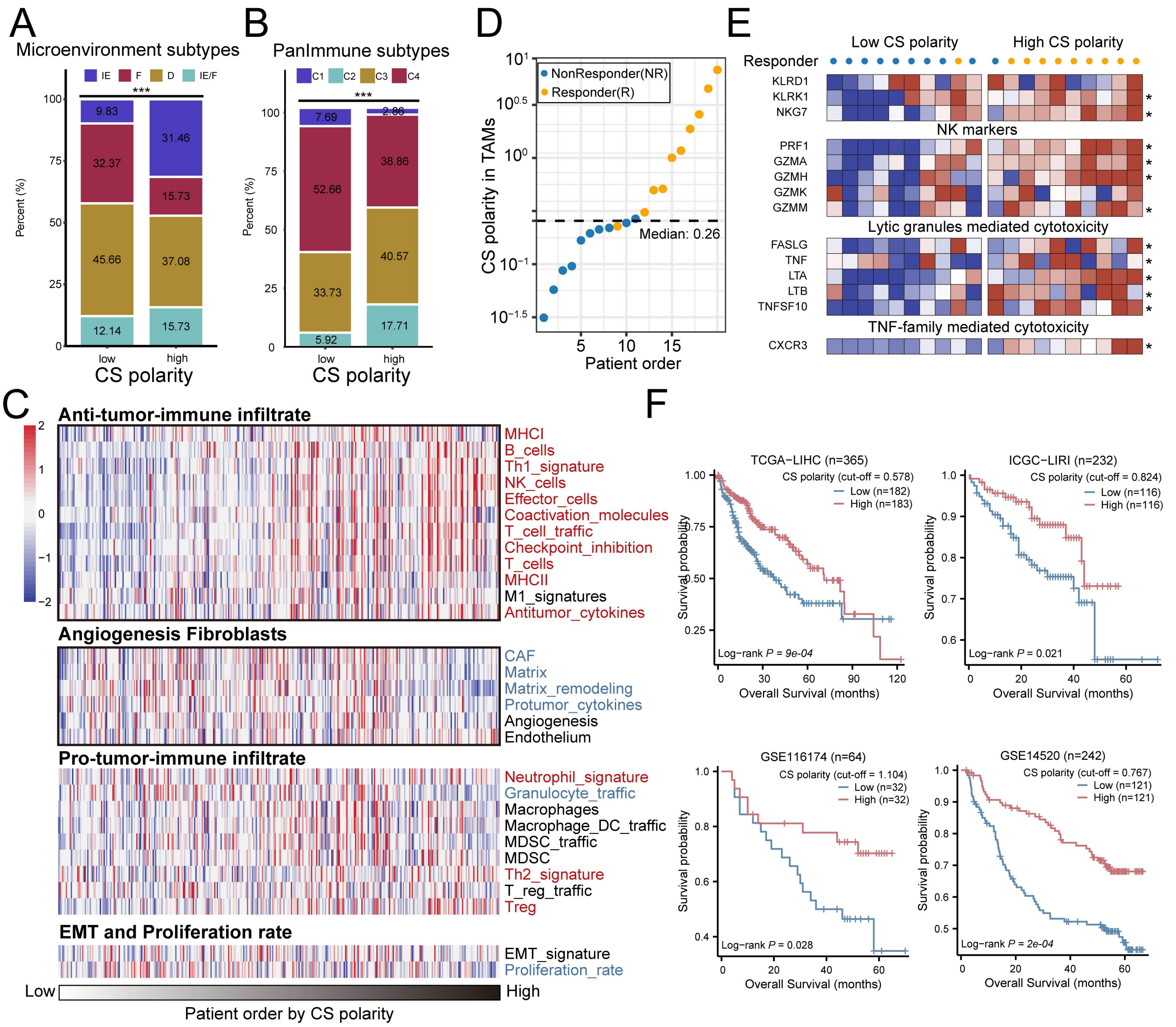

Supplementary Figure 7. The CS polarity could serve as a potential indicator of prognostic and microenvironmental status for HCC patients. (A, B) Bar graphs depicting the proportion (percentage) of patients from microenvironment subtyping (A) and PanImmune subtyping (B). (C) Heatmap of the Spearman correlations between the CS polarization level and immune functional gene sets. (D) Patient ranking according to the CS polarity in the scRNA-seq dataset on immunotherapy. The dashed line represents median of the CS polarity in the scRNA-seq dataset on immunotherapy. (E) Heatmaps showing expression of cytotoxic genes in patients ordered by the CS polarity in (D). (F) Kaplan-Meier analysis showing an association of worse overall survival with lower CS polarity in four HCC cohorts.

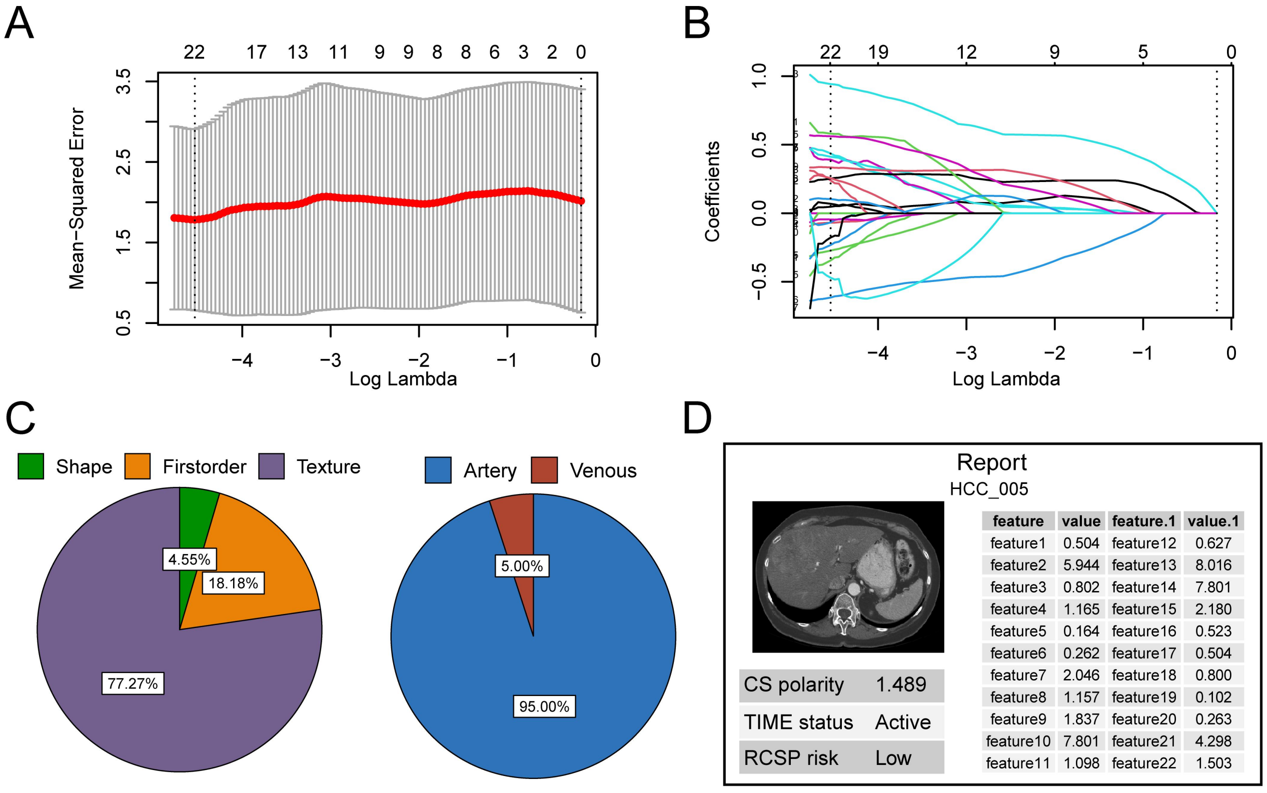

Supplementary Figure 8. CT-based radiomics model non-invasively predicted the CS polarity in HCC patients. (A) Parameter tuning plot in least absolute shrinkage and selection operator (LASSO) regression. The topmost values represent the number of radiomics features screened by Pearson's correlation that are incorporated into the LASSO regression model. λ, the weight of the L1 norm. The two vertical dashed lines represent λ_{min} and λ_{1se}. λ_{min}, the value of λ that results in the smallest mean value of the squared error of the regression model. λ_{1se}, the value of λ that results in the simplest model within an SE of λ_{min}. (B) Distribution of coefficients for variables in the LASSO regression. Each curve represents a radiomics feature filtered by Pearson correlation. The y-axis is their corresponding coefficient in the LASSO regression. The top most values carry the same meaning as depicted in (A). (C) The pie charts indicate the distribution of selected radiomics features in classes and phases. (D) A report of the CS polarity prediction is generated for given images of patients.
